# Supplementary figures and images for: CCDC113 stabilizes sperm axoneme and head-tail coupling apparatus to ensure male fertility
Source: eLife. 2024 Dec 13;13:RP98016. doi: 10.7554/eLife.98016 (PMC11643634; doi:10.7554/eLife.98016)

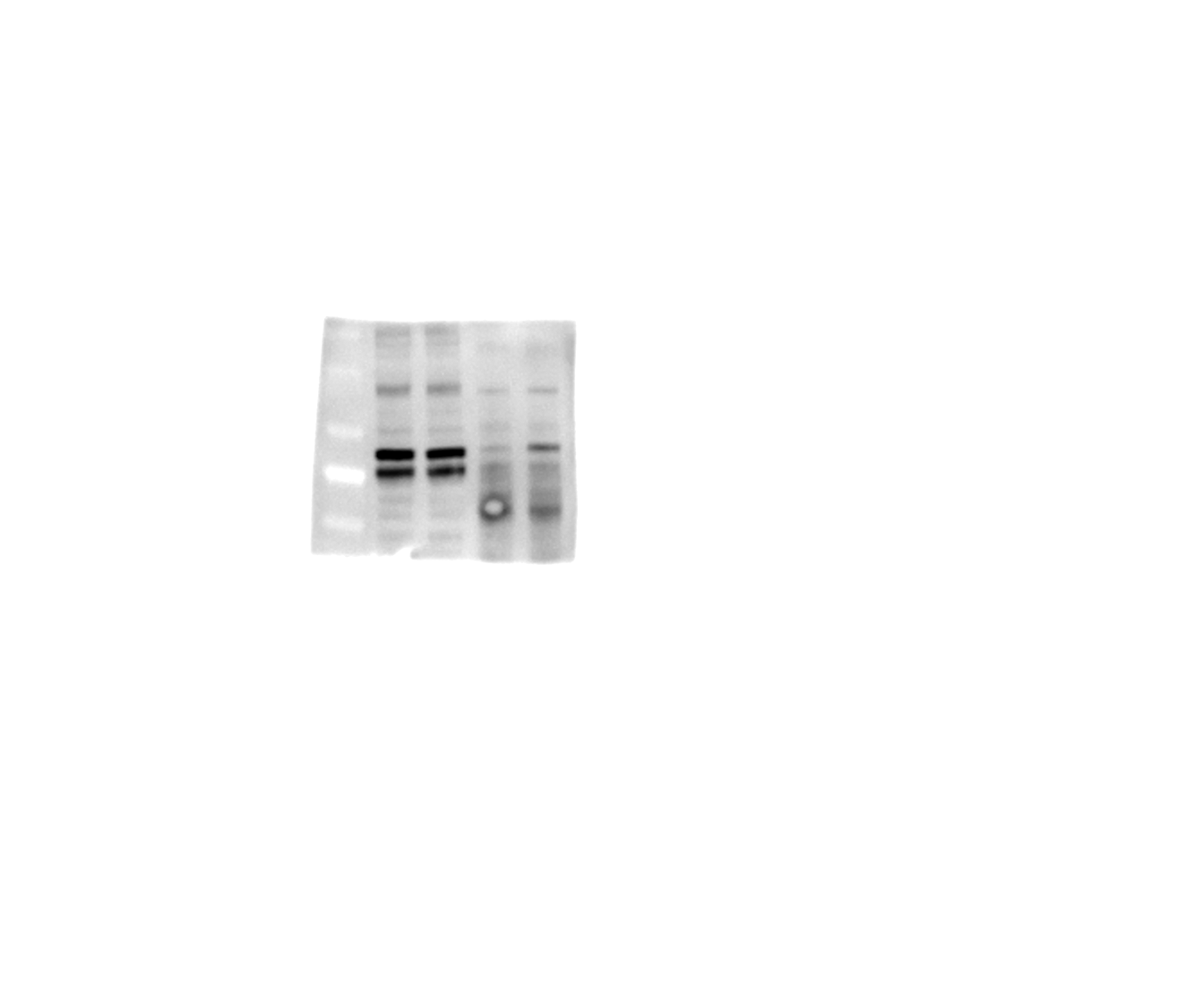

Supplement: Figure 1—source data 1. [file elife-98016-fig1-data1.zip › Figure 1-Source Data 1/Figure 1E GFP CCDC113 unedited.Tif]

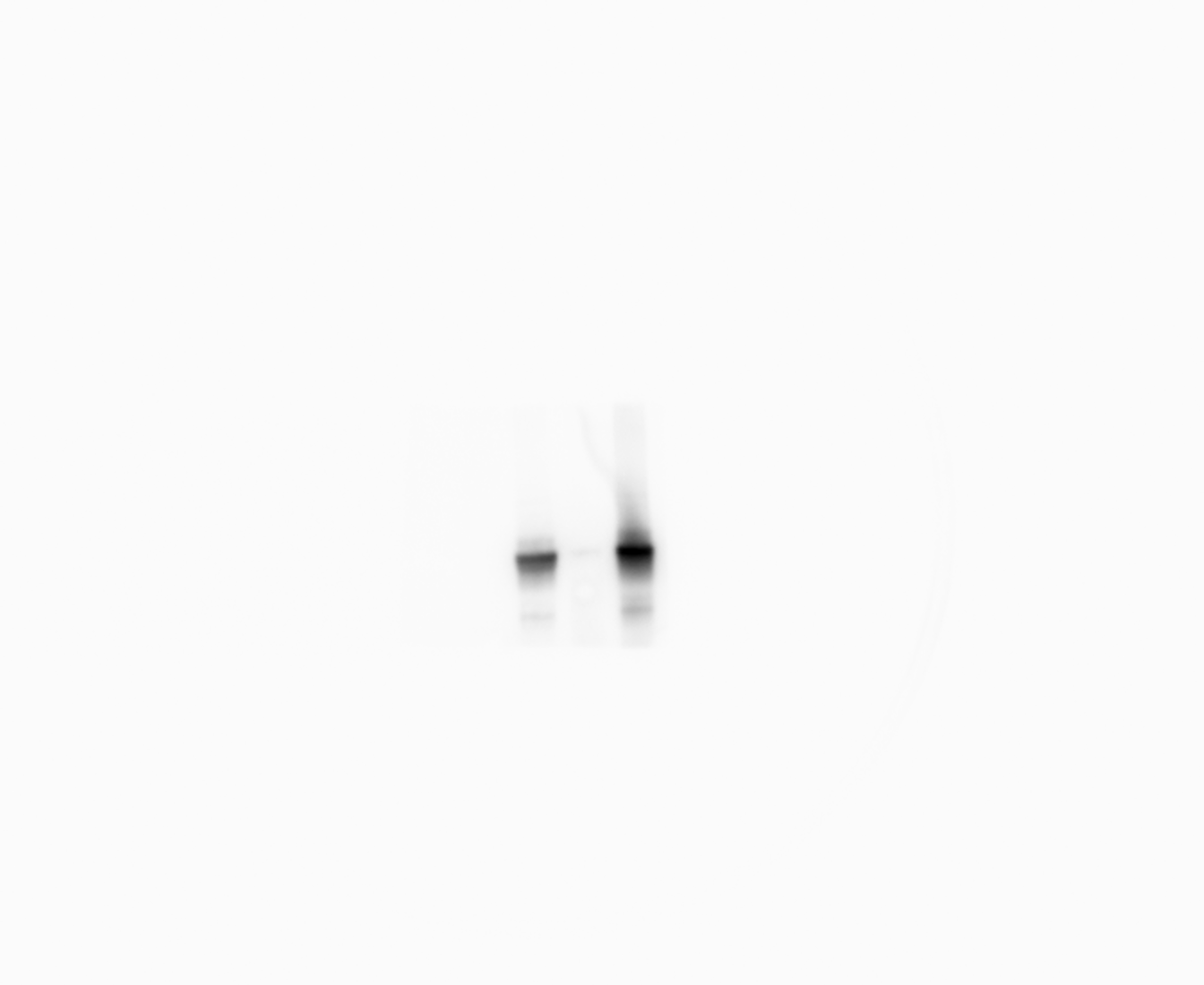

Supplement: Figure 1—source data 1. [file elife-98016-fig1-data1.zip › Figure 1-Source Data 1/Figure 1E MYC TUBB5 unedited.Tif]

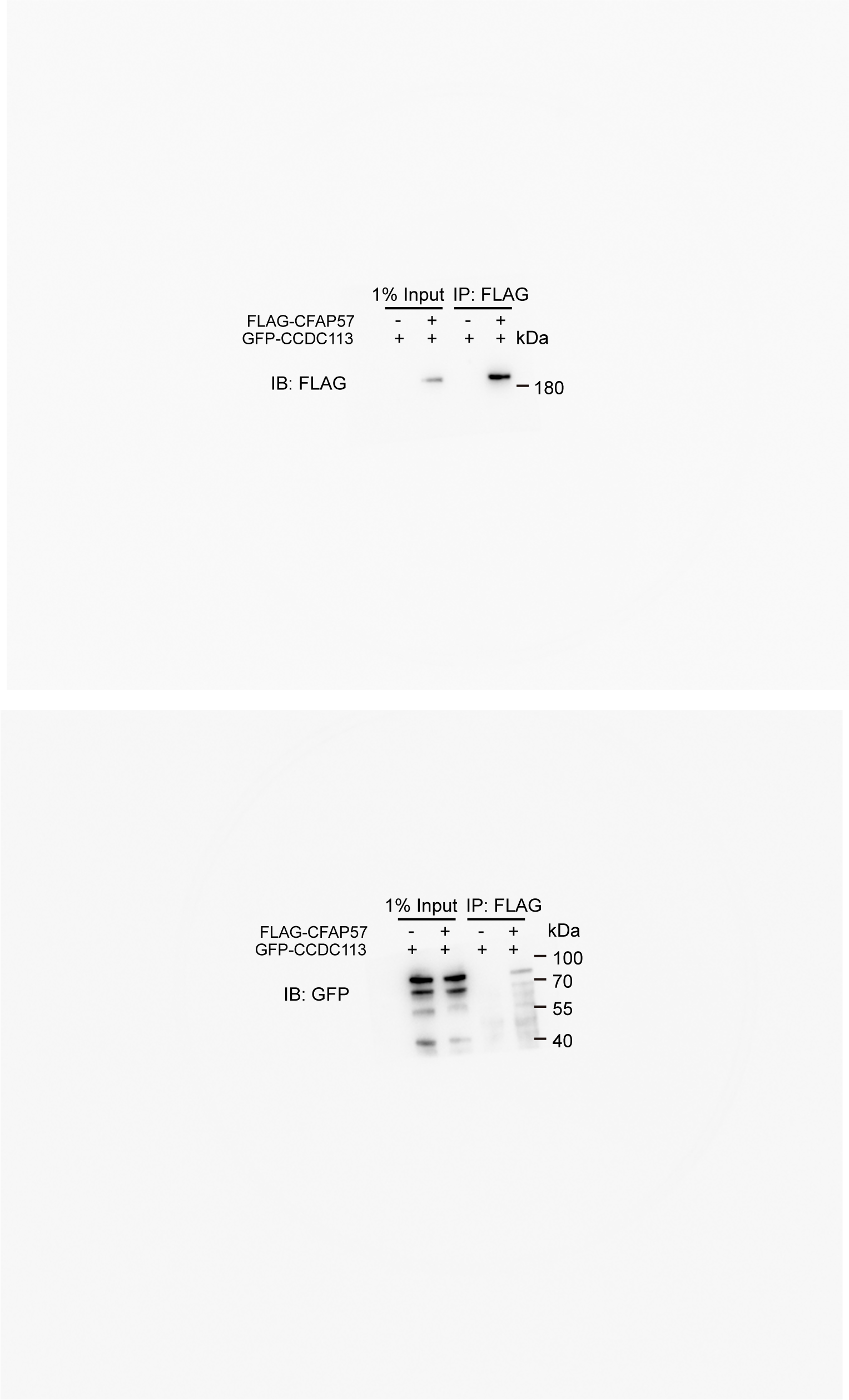

Supplement: Figure 1—source data 2. [file elife-98016-fig1-data2.zip › Figure 1-Source Data 2/Figure 1C labelled.tif]

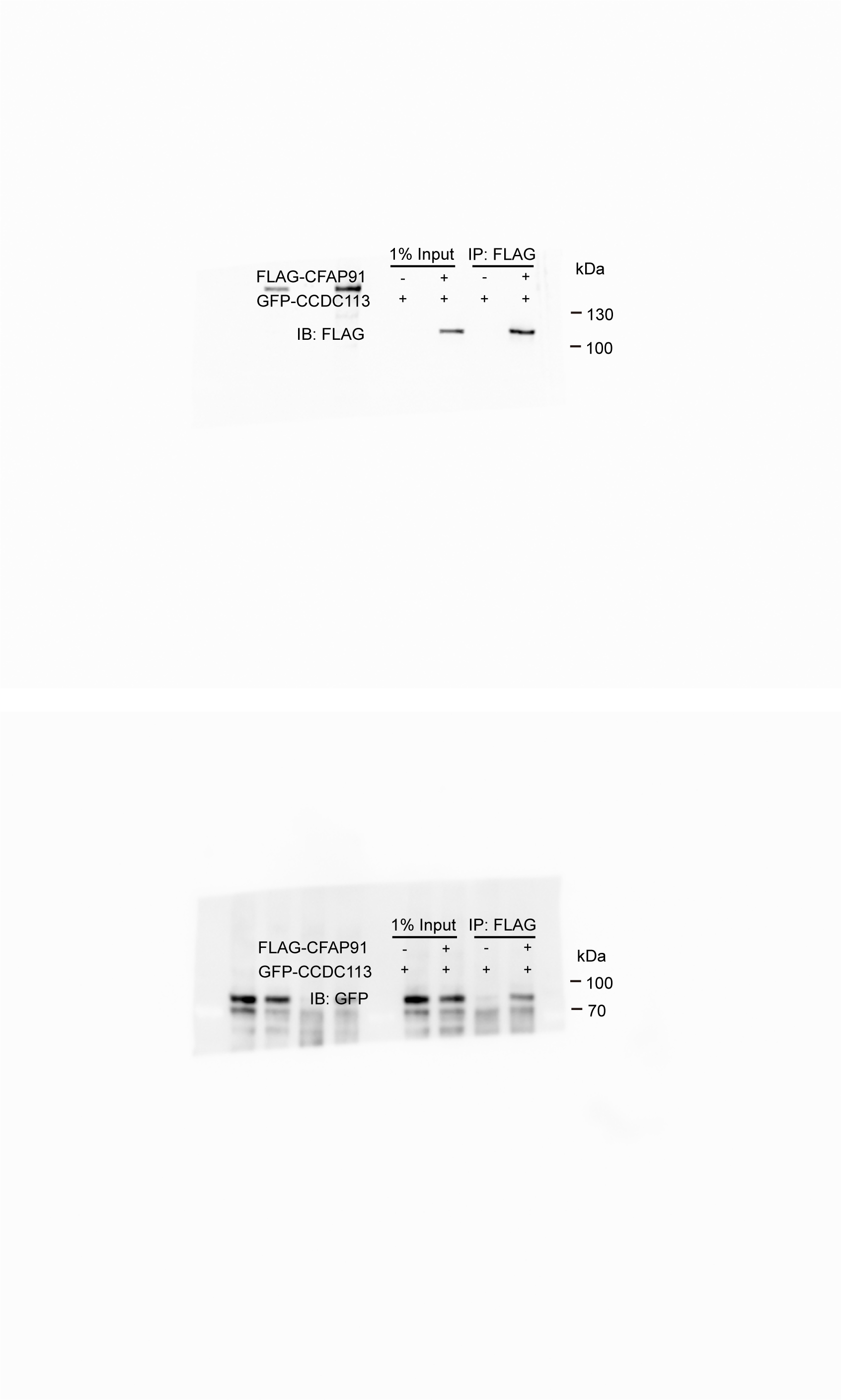

Supplement: Figure 1—source data 2. [file elife-98016-fig1-data2.zip › Figure 1-Source Data 2/Figure 1D labelled.tif]

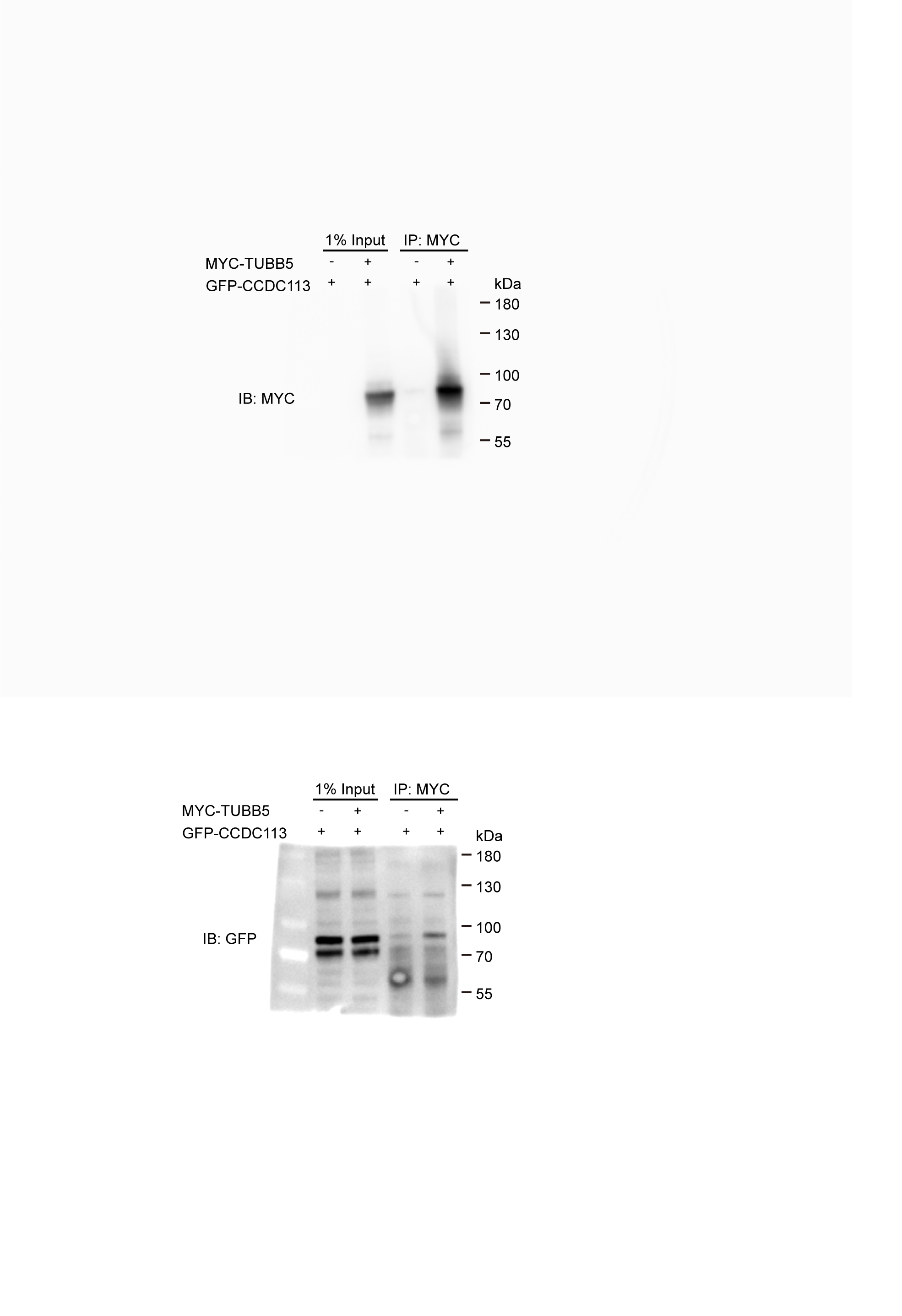

Supplement: Figure 1—source data 2. [file elife-98016-fig1-data2.zip › Figure 1-Source Data 2/Figure 1E labelled.tif]

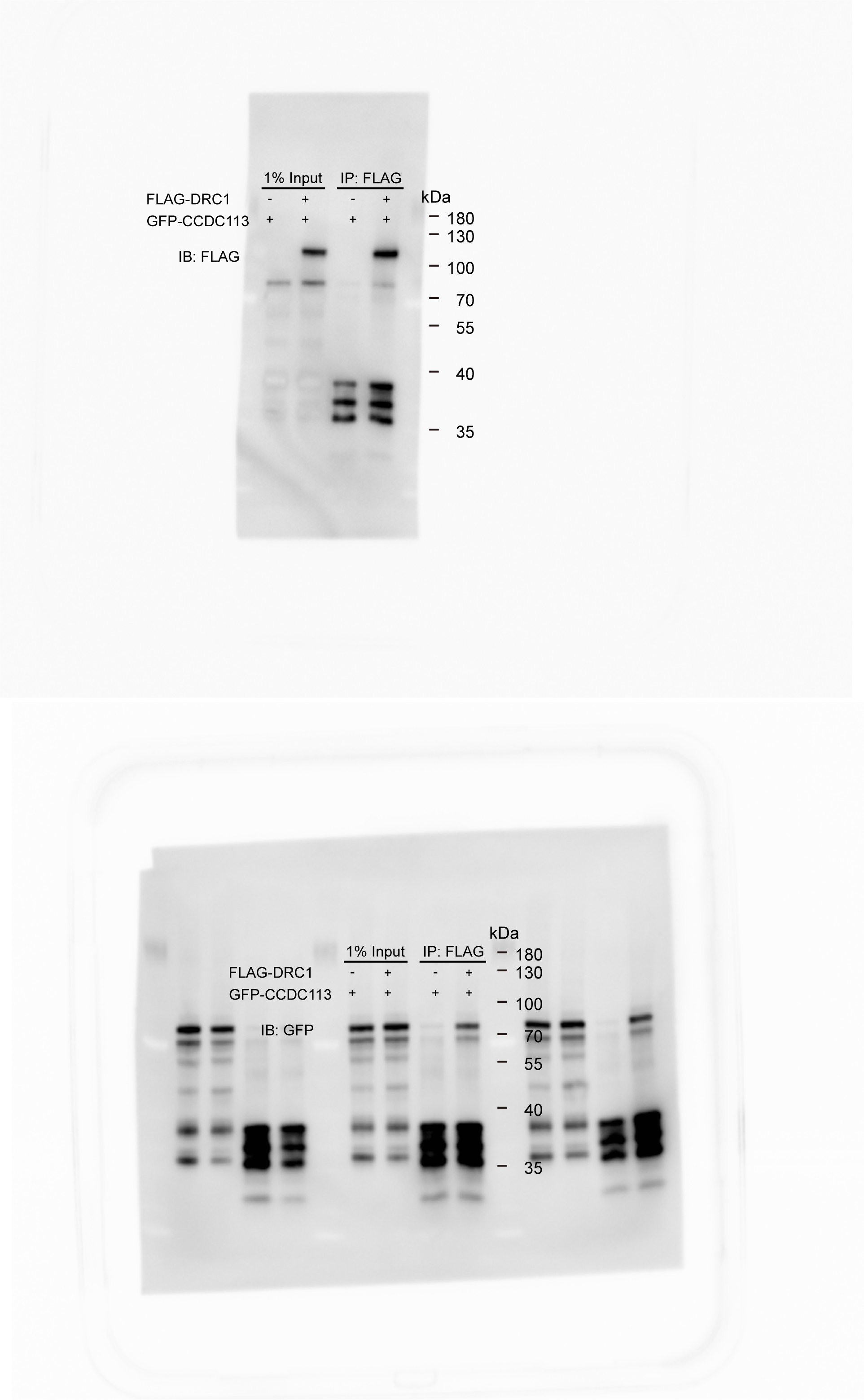

Supplement: Figure 1—source data 2. [file elife-98016-fig1-data2.zip › Figure 1-Source Data 2/figure 1F labelled.tif]

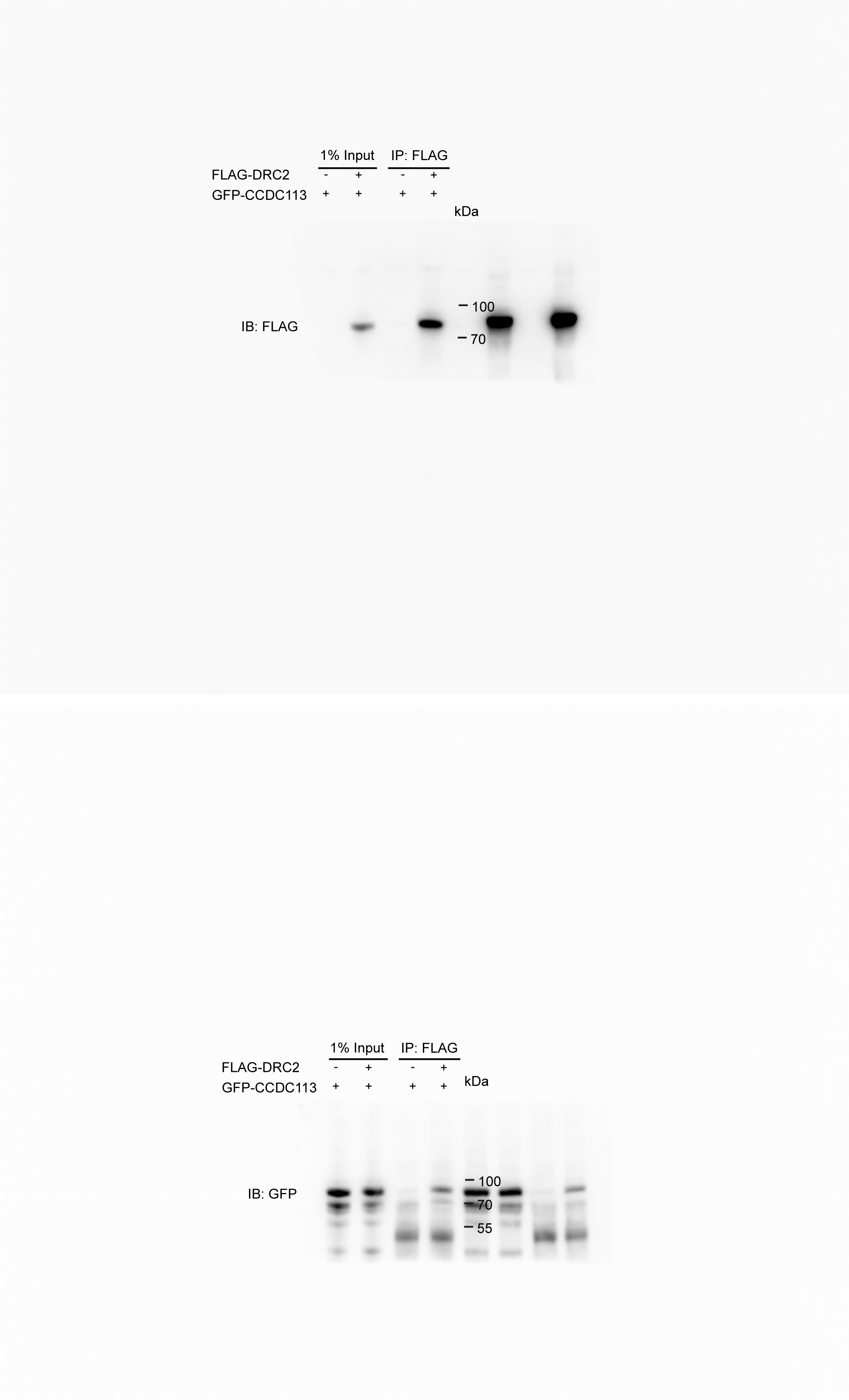

Supplement: Figure 1—source data 2. [file elife-98016-fig1-data2.zip › Figure 1-Source Data 2/figure 1G labelled.tif]

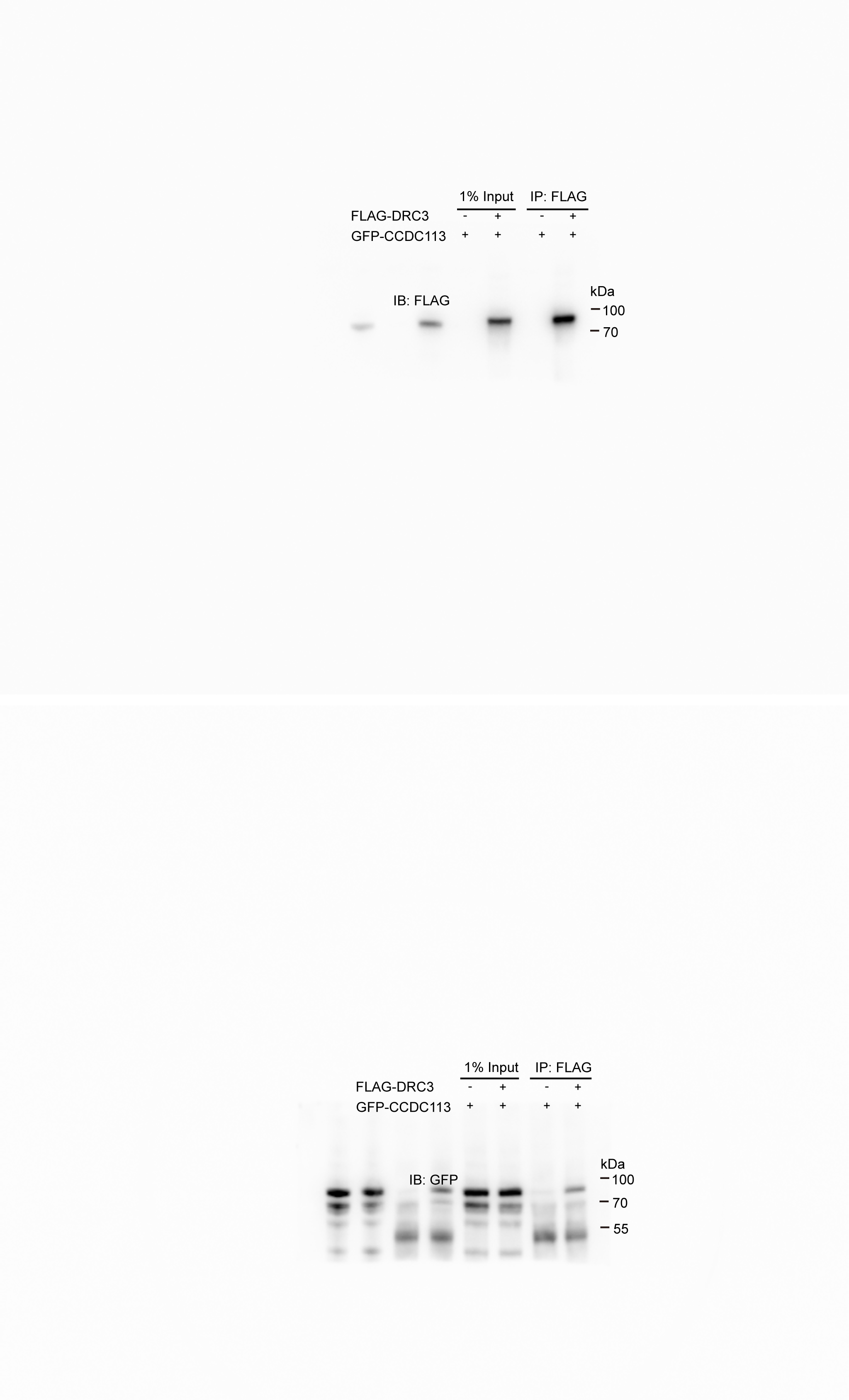

Supplement: Figure 1—source data 2. [file elife-98016-fig1-data2.zip › Figure 1-Source Data 2/figure 1H labelled.tif]

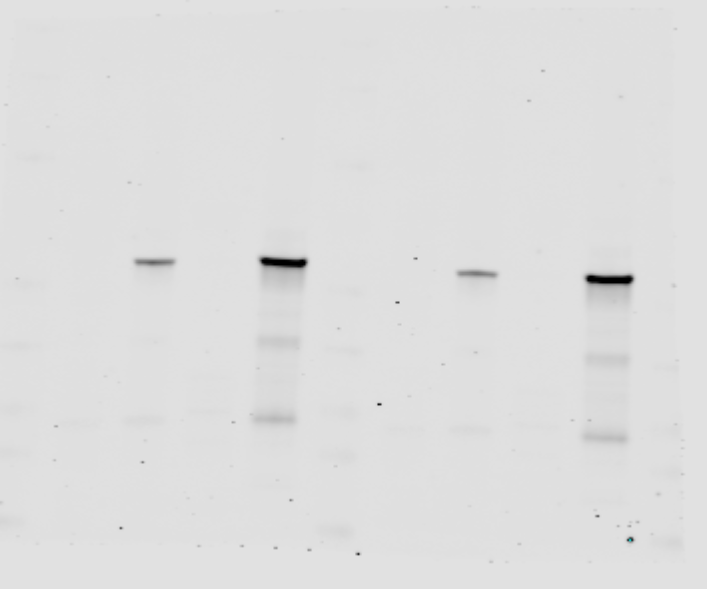

Supplement: Figure 1—figure supplement 1—source data 1. [file elife-98016-fig1-figsupp1-data1.zip › Figure 1-Figure Supplement 1-Source Data 1/Figure 1A FLAG CCDC113-unedited.tif]

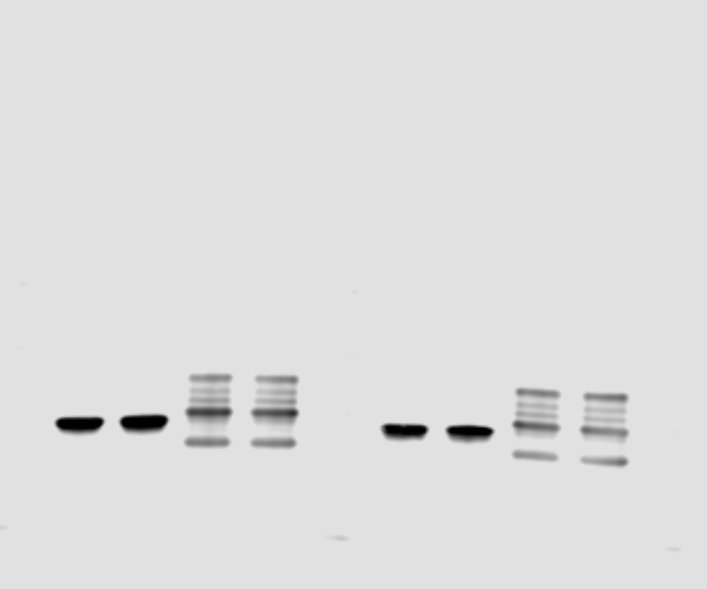

Supplement: Figure 1—figure supplement 1—source data 1. [file elife-98016-fig1-figsupp1-data1.zip › Figure 1-Figure Supplement 1-Source Data 1/Figure 1A GFP ODF1-unedited.tif]

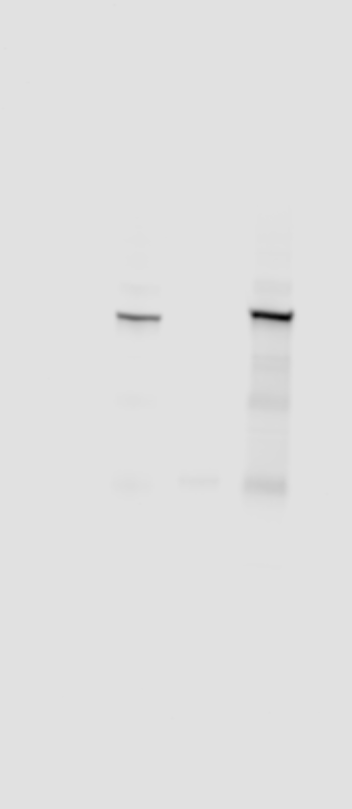

Supplement: Figure 1—figure supplement 1—source data 1. [file elife-98016-fig1-figsupp1-data1.zip › Figure 1-Figure Supplement 1-Source Data 1/Figure 1B FLAG CCDC113-unedited.tif]

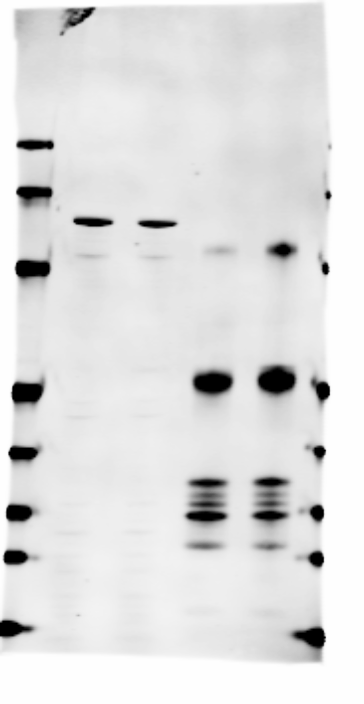

Supplement: Figure 1—figure supplement 1—source data 1. [file elife-98016-fig1-figsupp1-data1.zip › Figure 1-Figure Supplement 1-Source Data 1/Figure 1B HA ODF2-unedited.tif]

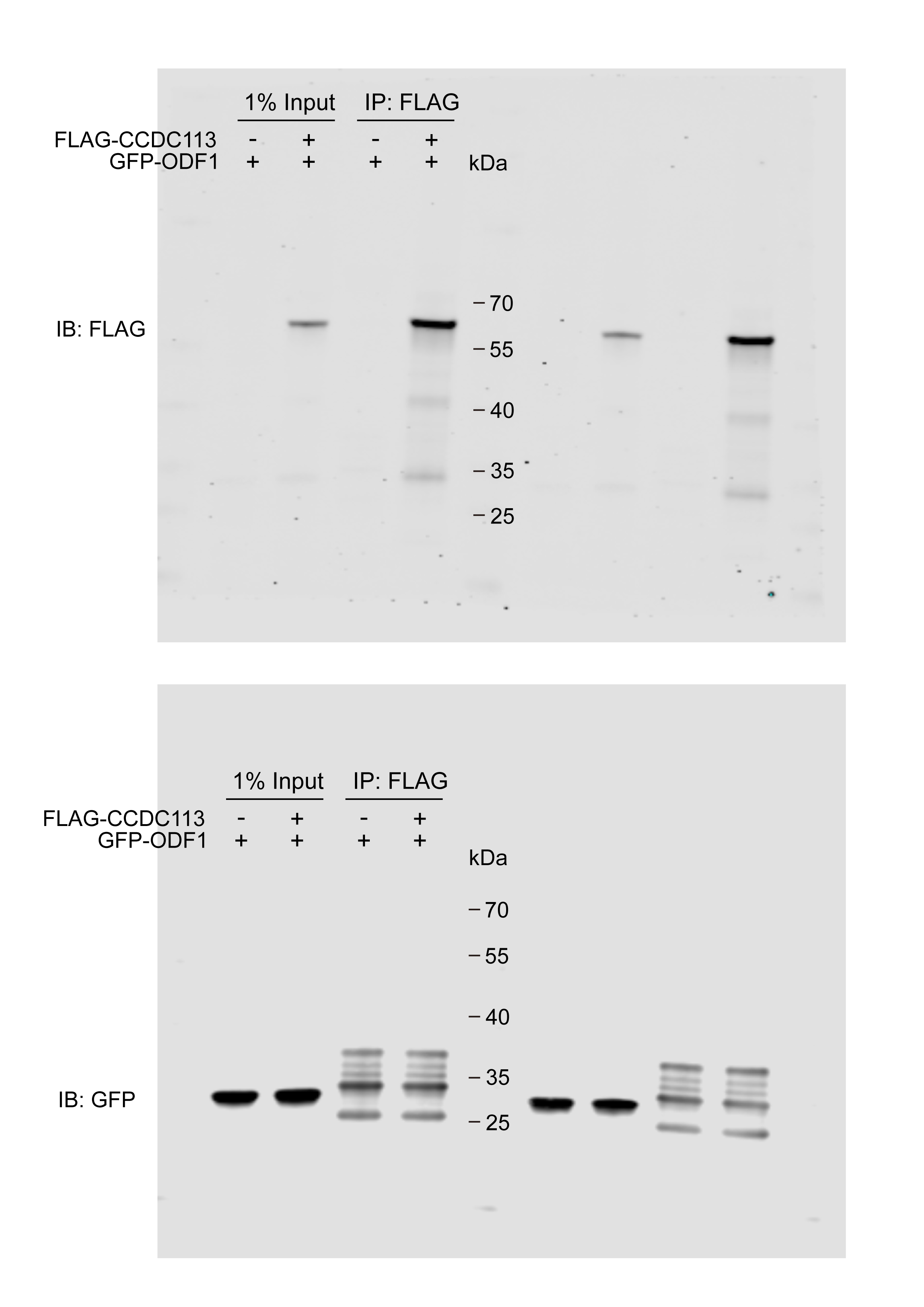

Supplement: Figure 1—figure supplement 1—source data 2. [file elife-98016-fig1-figsupp1-data2.zip › Figure 1-Figure Supplement 1-Source Data 2/Figure 1A labelled.tif]

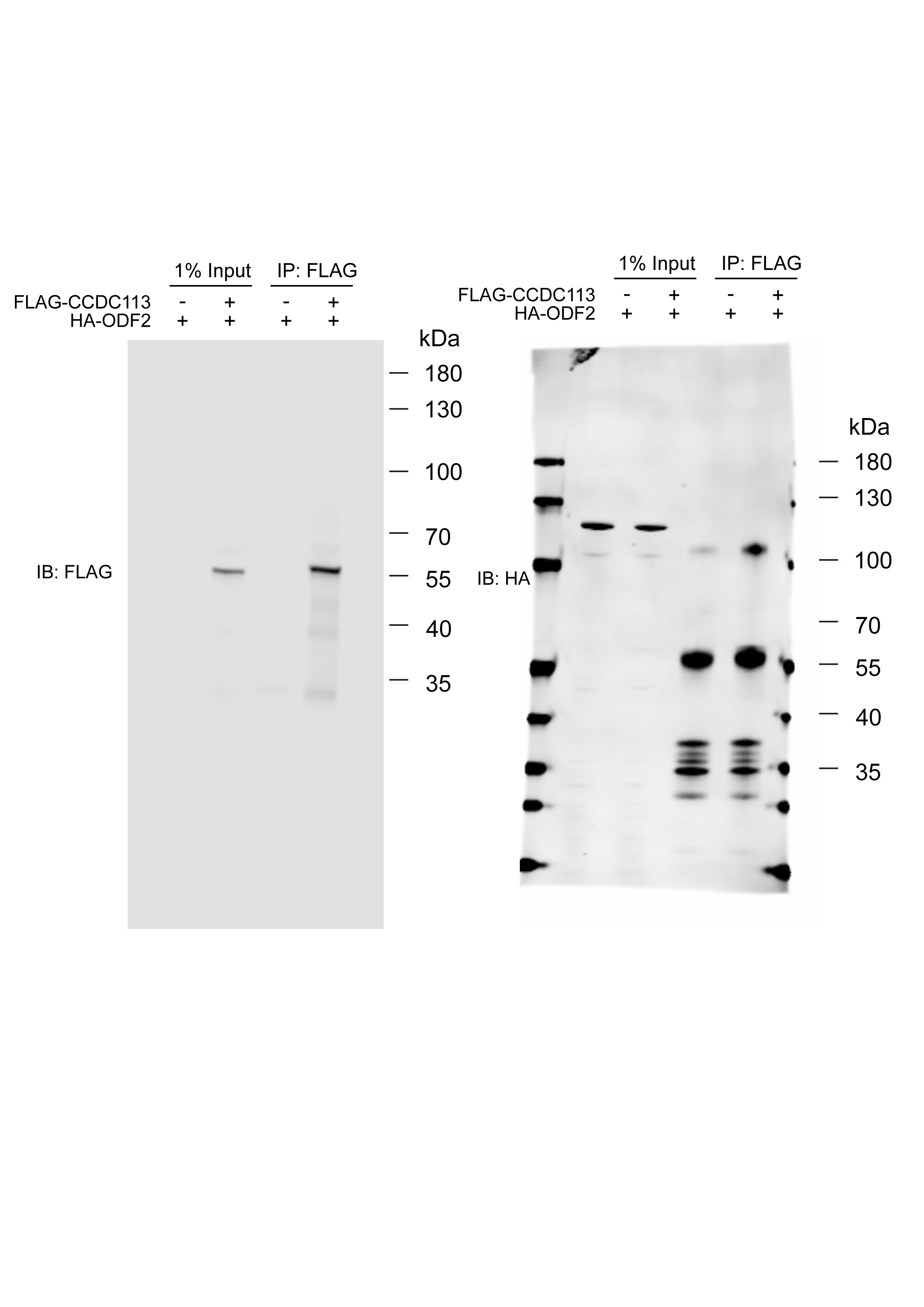

Supplement: Figure 1—figure supplement 1—source data 2. [file elife-98016-fig1-figsupp1-data2.zip › Figure 1-Figure Supplement 1-Source Data 2/Figure 1B labelled.tif]

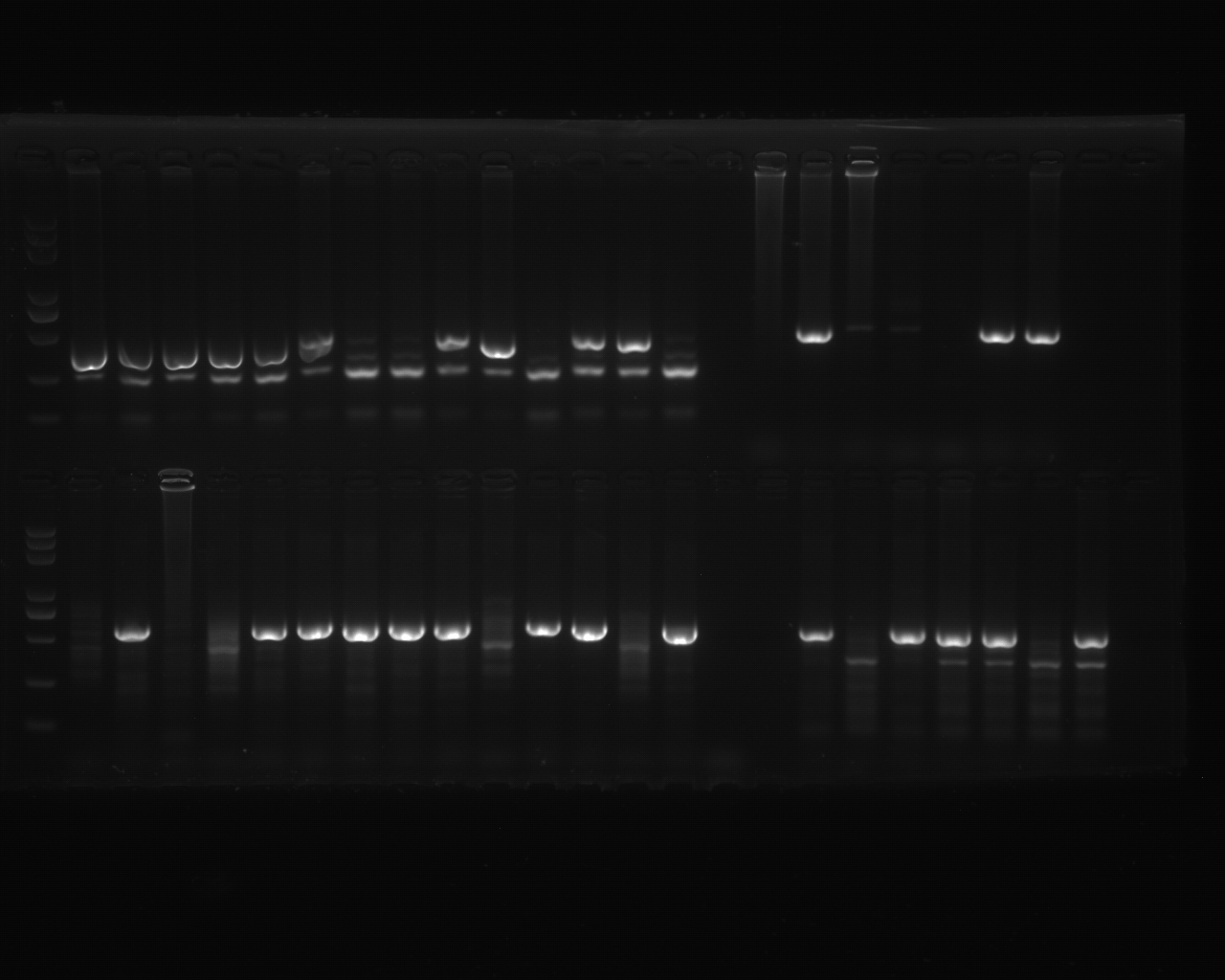

Supplement: Figure 2—source data 1. [file elife-98016-fig2-data1.zip › Figure 2-Source Data 1/Figure 2B unedited raw data.bmp]

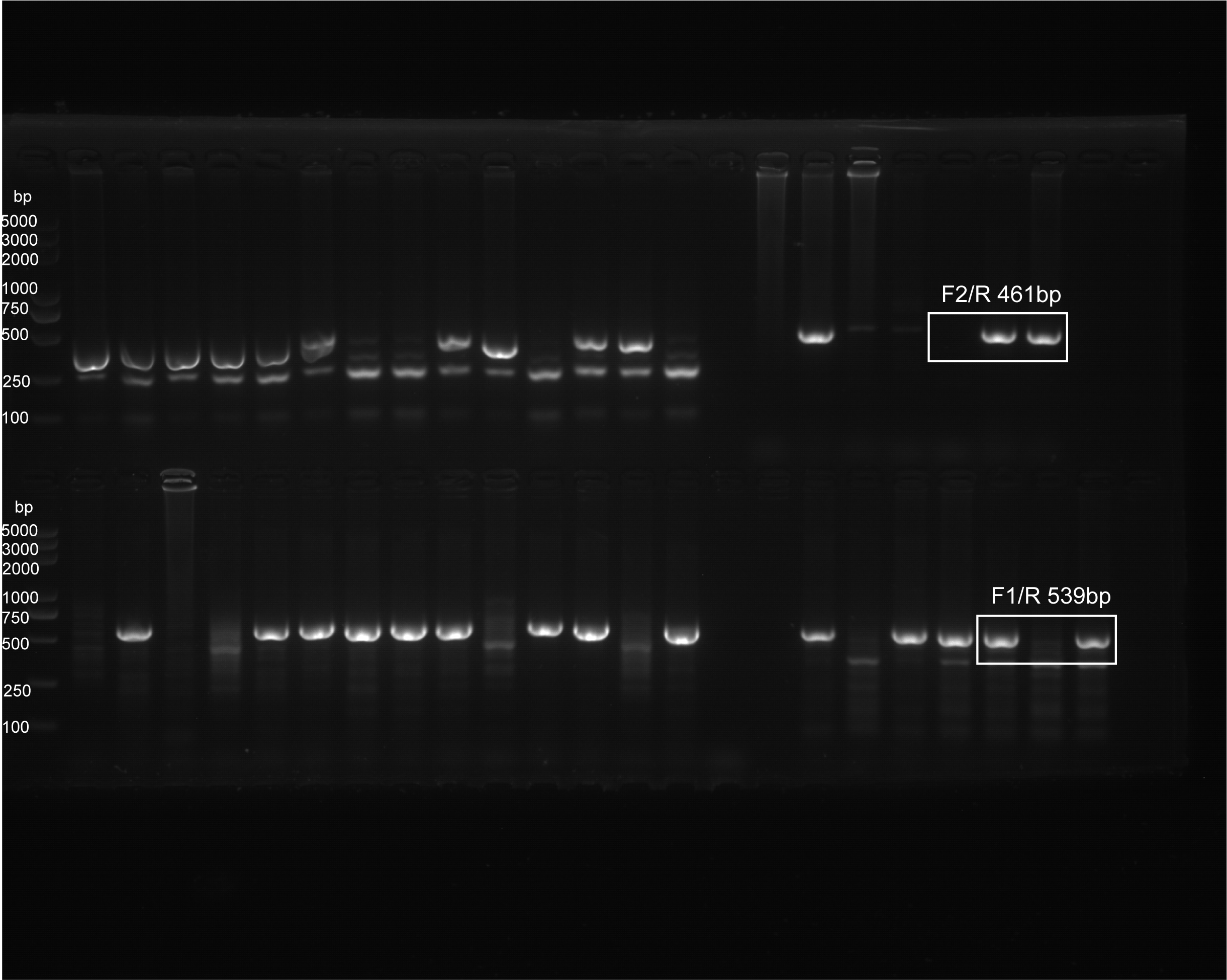

Supplement: Figure 2—source data 2. [file elife-98016-fig2-data2.zip › Figure 2-Source Data 2/Figure 2B labelled.tif]

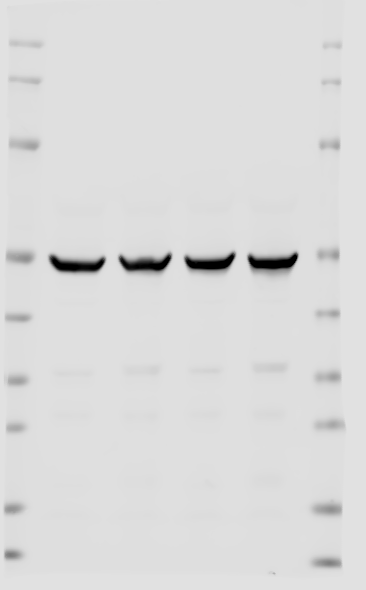

Supplement: Figure 2—source data 3. [file elife-98016-fig2-data3.zip › Figure 2-Source Data 3/Figure 2C CCDC113 WT KO TESTIS TUBULIN-unedited.tif]

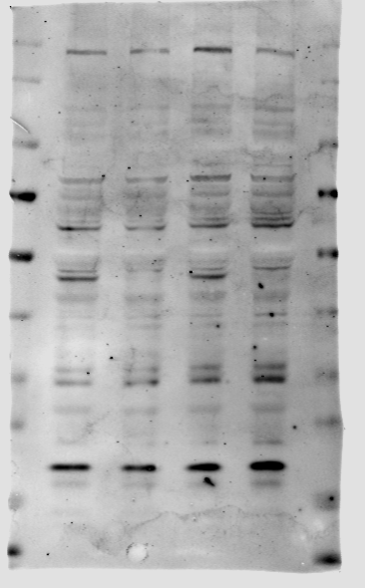

Supplement: Figure 2—source data 3. [file elife-98016-fig2-data3.zip › Figure 2-Source Data 3/Figure 2C CCDC113 WT KO TESTIS-unedited.tif]

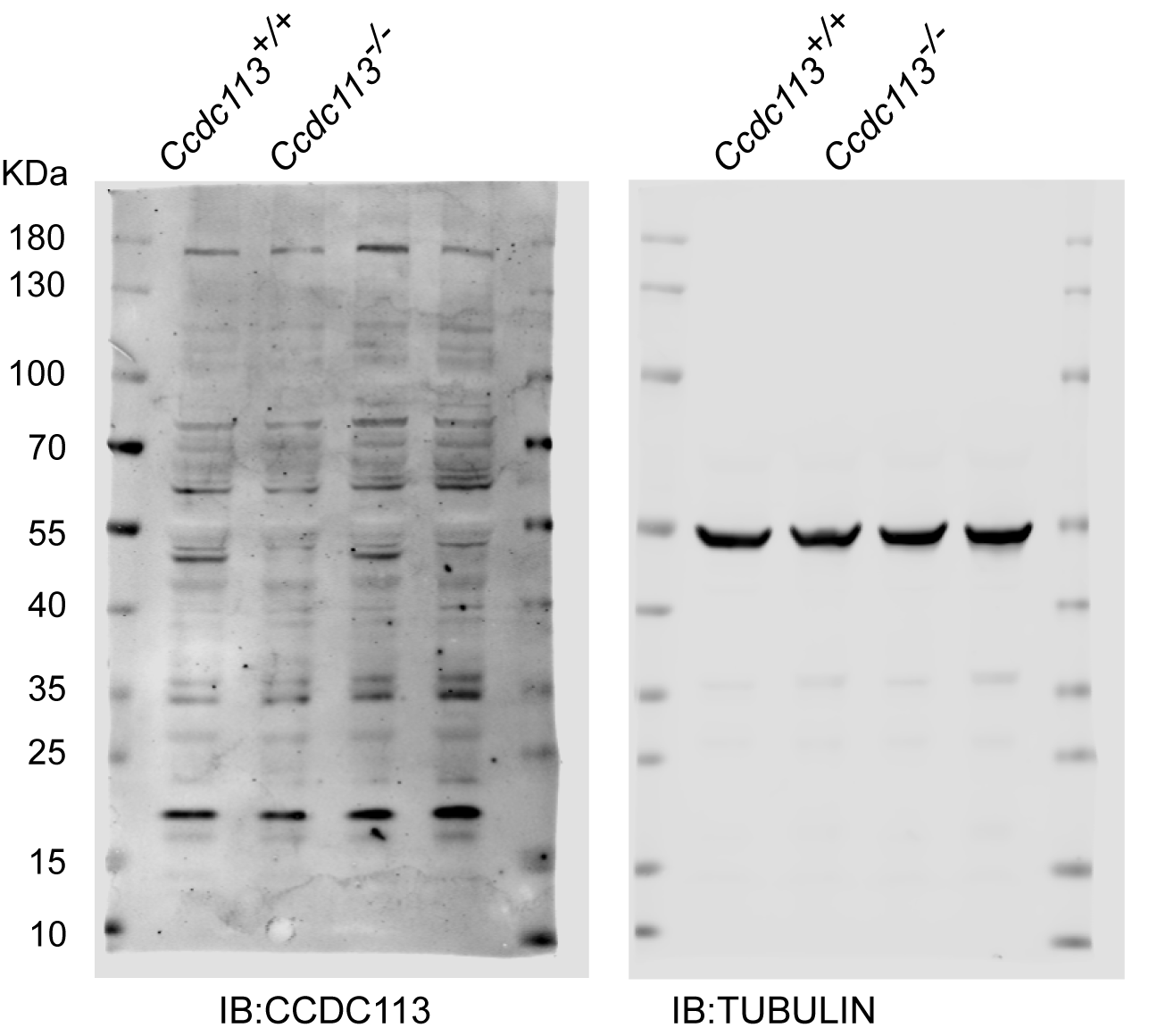

Supplement: Figure 2—source data 4. [file elife-98016-fig2-data4.zip › Figure 2-Source Data 4/Figure 2C labelled.tif]

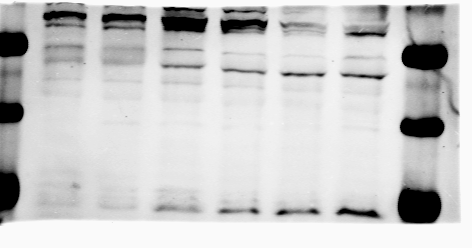

Supplement: Figure 4—source data 1. [file elife-98016-fig4-data1.zip › Figure 4-Source Data 1/CCDC113 P7 P14 P21 P28 P35 P56 unedited.tif]

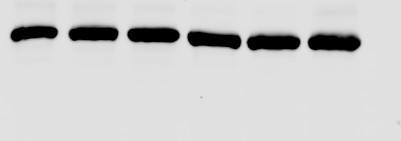

Supplement: Figure 4—source data 1. [file elife-98016-fig4-data1.zip › Figure 4-Source Data 1/tubulin unedited.tif]

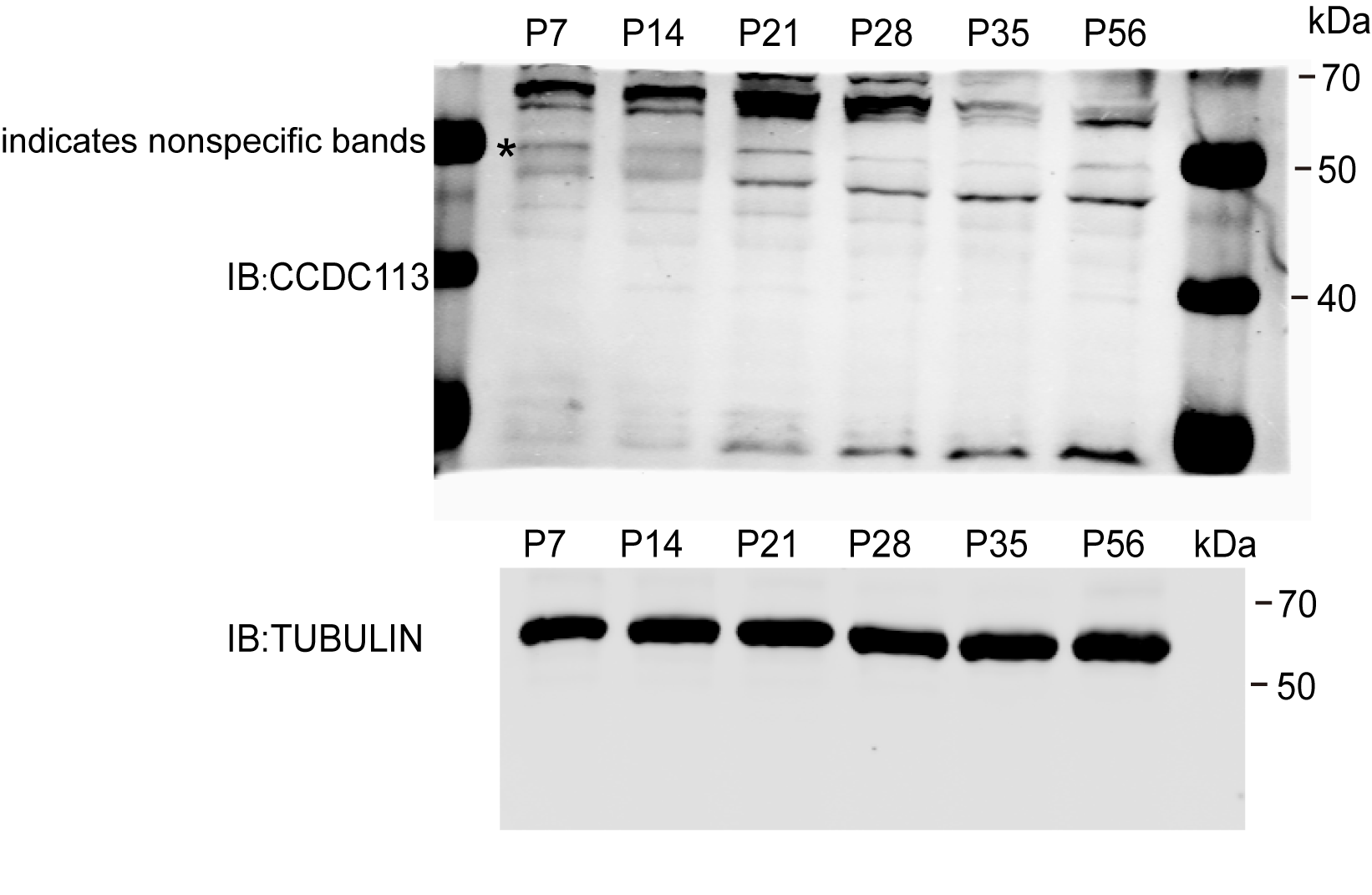

Supplement: Figure 4—source data 2. [file elife-98016-fig4-data2.zip › Figure 4-Source Data 2/Figure 4A labelled.tif]

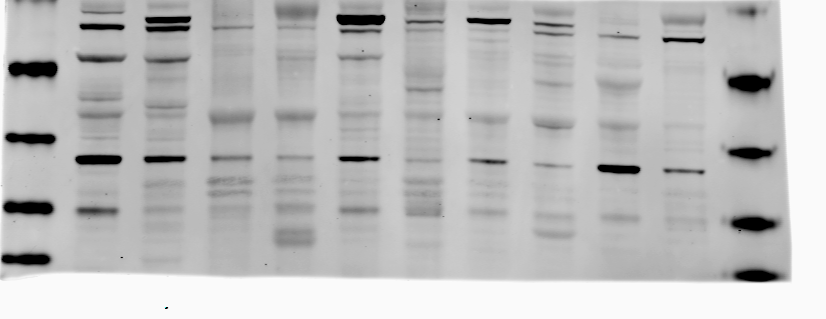

Supplement: Figure 4—figure supplement 1—source data 1. [file elife-98016-fig4-figsupp1-data1.zip › Figure 4-Figure Supplement 1-Source Data 1/Figure 4B CCDC113 unedited.tif]

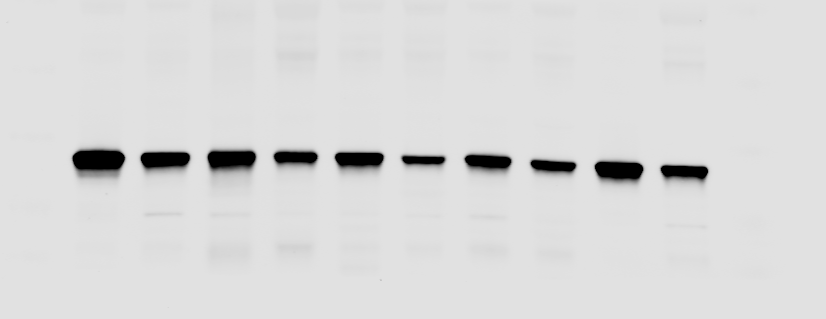

Supplement: Figure 4—figure supplement 1—source data 1. [file elife-98016-fig4-figsupp1-data1.zip › Figure 4-Figure Supplement 1-Source Data 1/Figure 4B GAPDH unedited.tif]

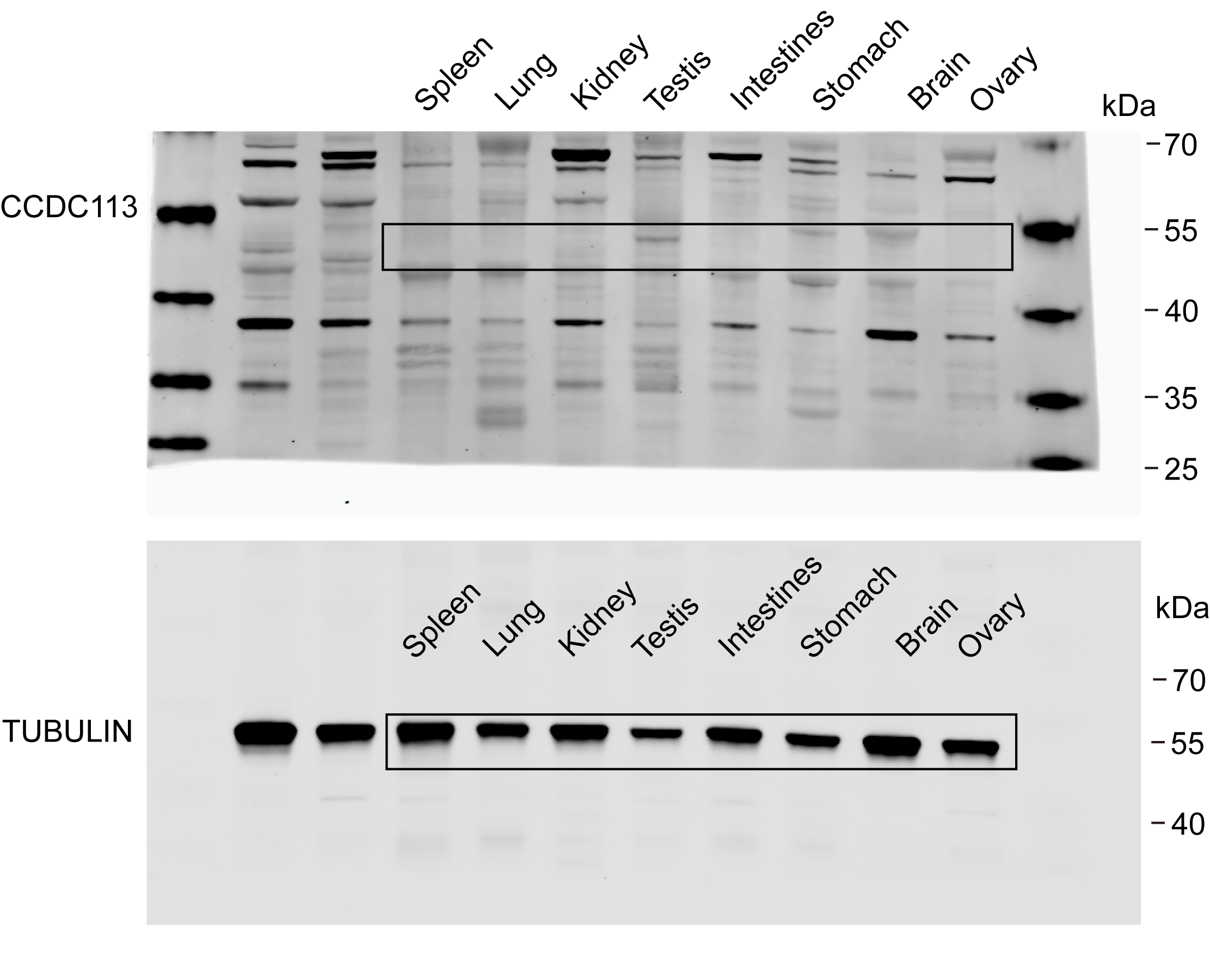

Supplement: Figure 4—figure supplement 1—source data 2. [file elife-98016-fig4-figsupp1-data2.zip › Figure 4-Figure Supplement 1-Source Data 2/Figure 4B labelled.tif]

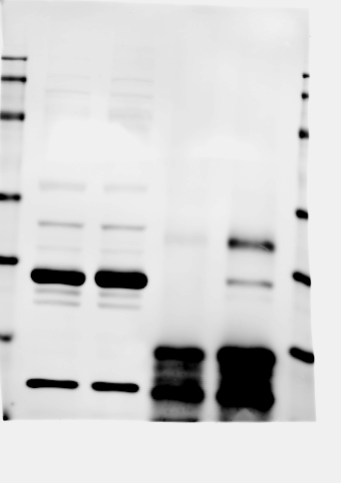

Supplement: Figure 7—source data 1. [file elife-98016-fig7-data1.zip › Figure 7-Source Data 1/Figure 7A FLAG SUN5- unedited.tif]

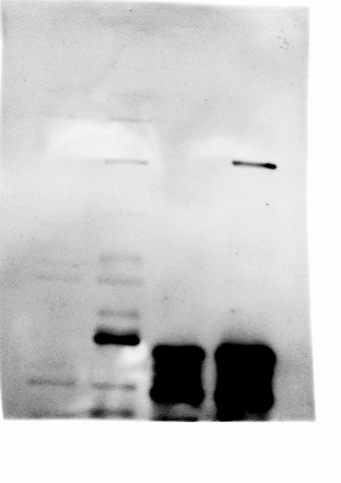

Supplement: Figure 7—source data 1. [file elife-98016-fig7-data1.zip › Figure 7-Source Data 1/Figure 7A GFP CCDC113-unedited.tif]

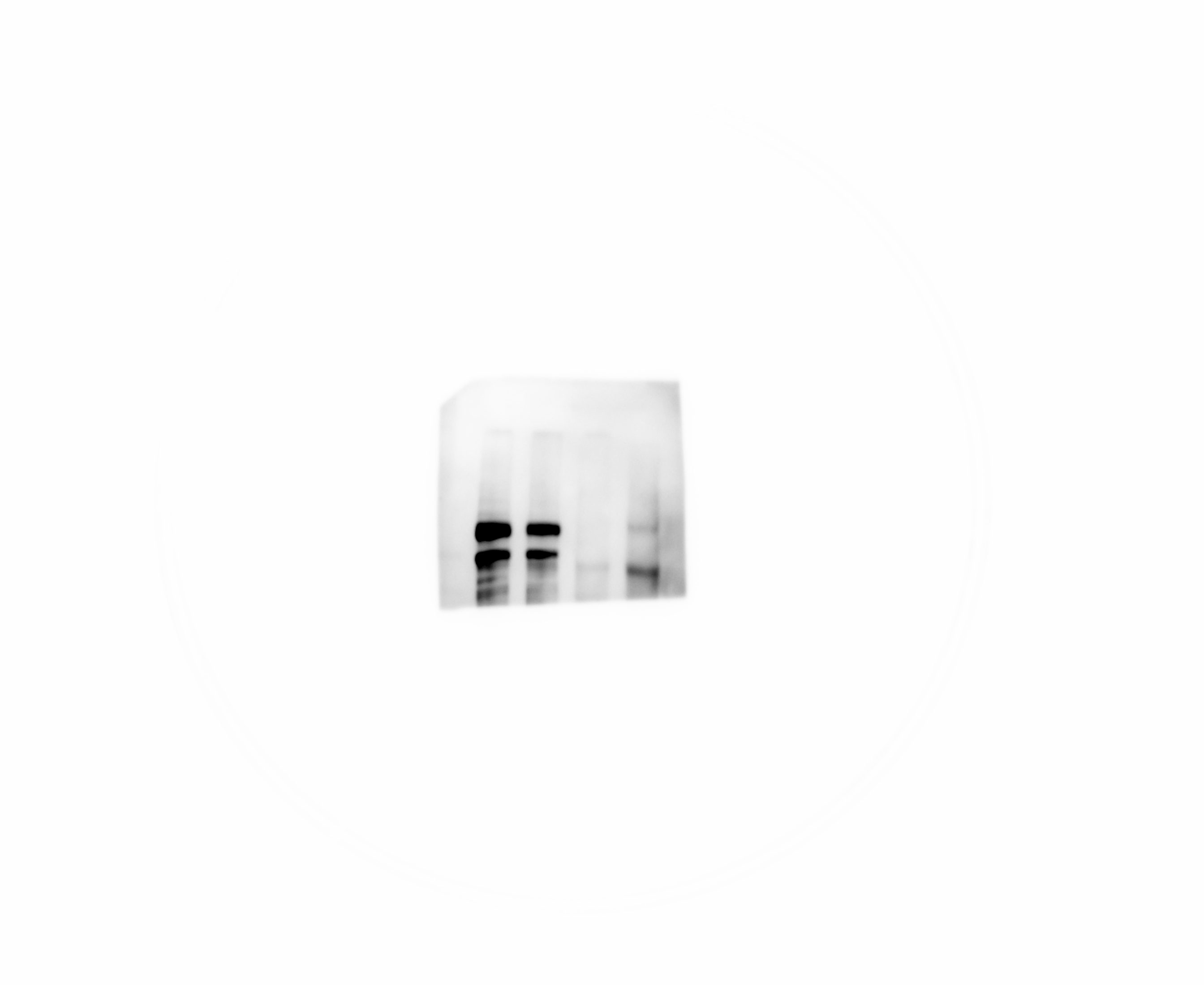

Supplement: Figure 7—source data 1. [file elife-98016-fig7-data1.zip › Figure 7-Source Data 1/Figure 7B FLAG CENTLEIN-unedited.Tif]

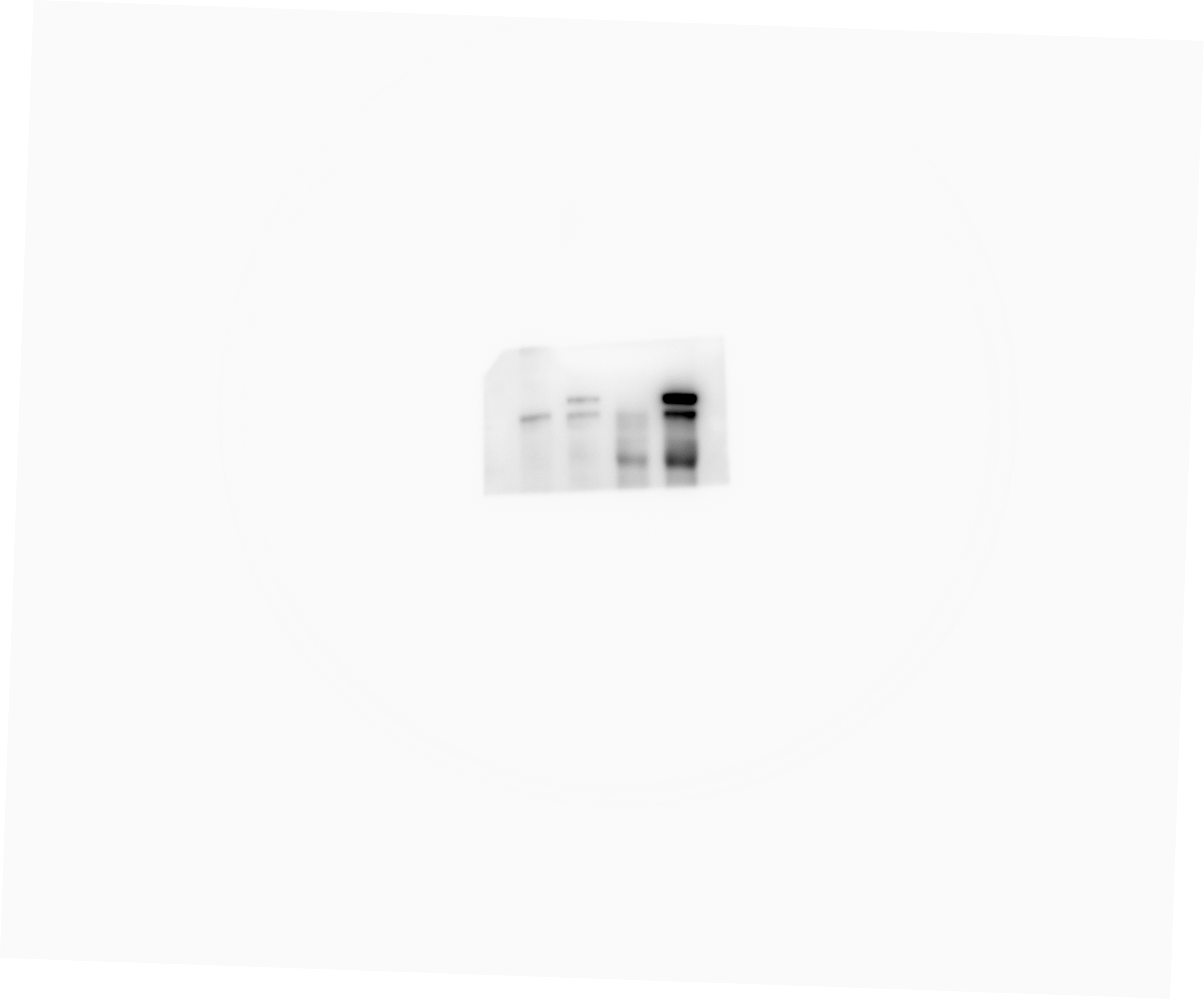

Supplement: Figure 7—source data 1. [file elife-98016-fig7-data1.zip › Figure 7-Source Data 1/Figure 7B GFP CCDC113-unedited.Tif]

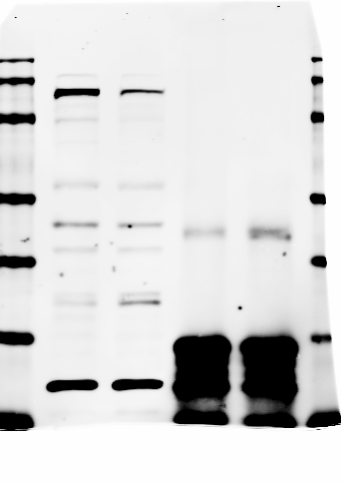

Supplement: Figure 7—source data 1. [file elife-98016-fig7-data1.zip › Figure 7-Source Data 1/Figure 7C FLAG PMFBP1-unedited.tif]

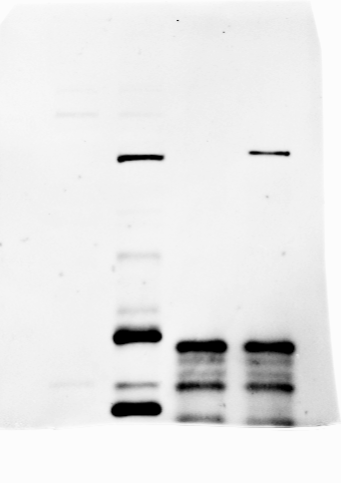

Supplement: Figure 7—source data 1. [file elife-98016-fig7-data1.zip › Figure 7-Source Data 1/Figure 7C GFP CCDC113-unedited.tif]

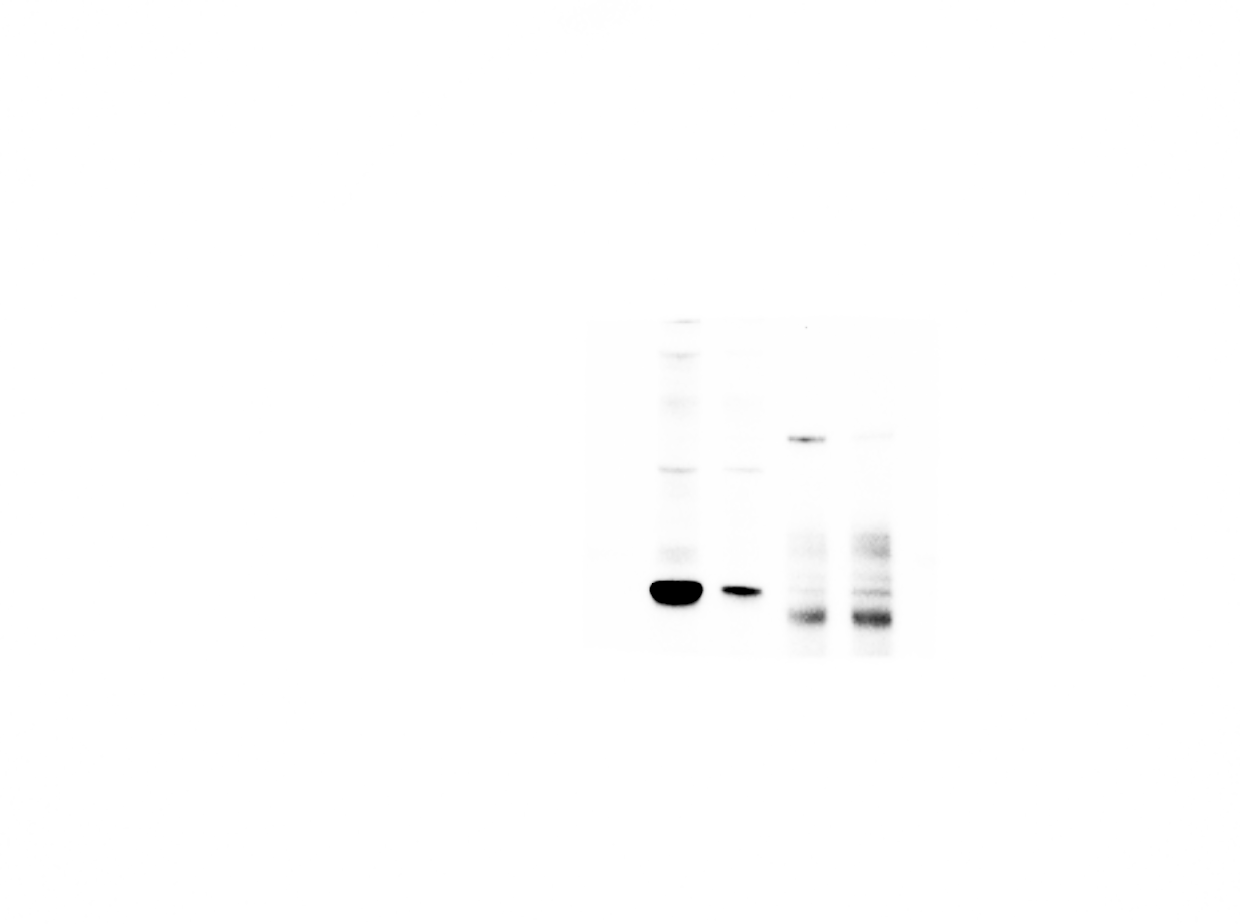

Supplement: Figure 7—source data 1. [file elife-98016-fig7-data1.zip › Figure 7-Source Data 1/Figure 7D FLAG CCDC113-unedited.tif]

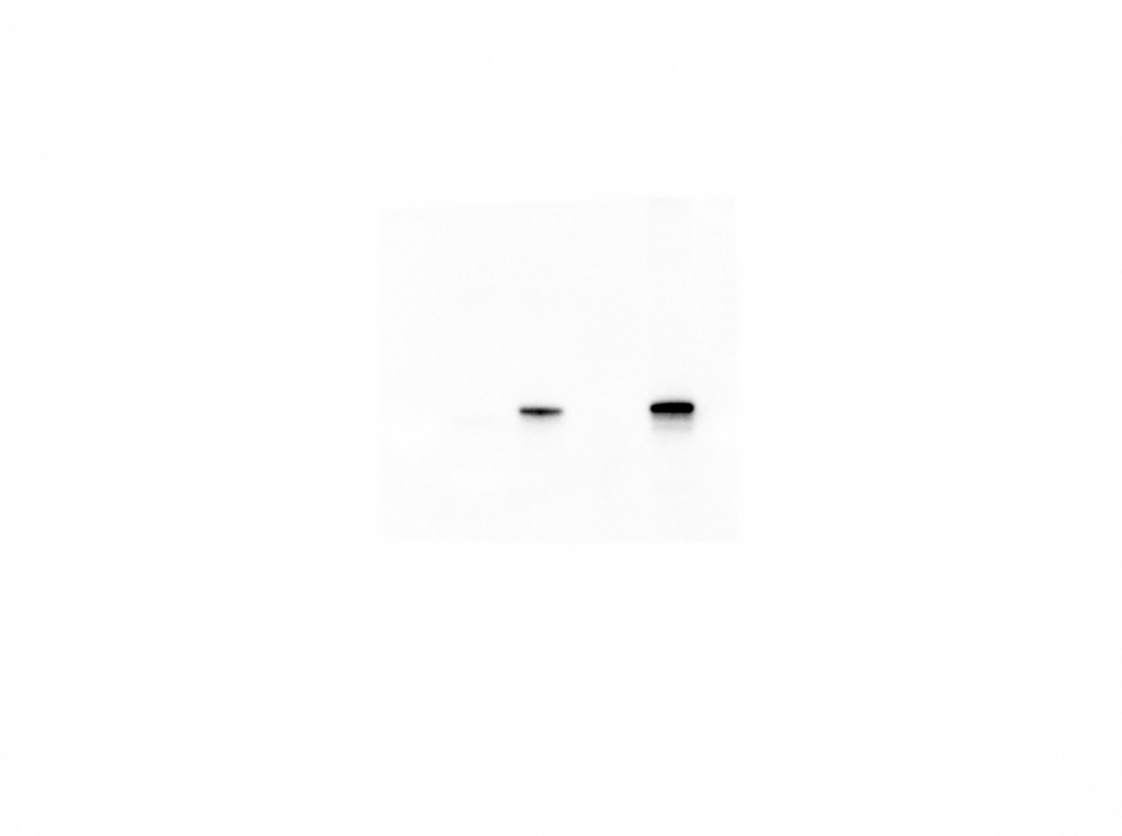

Supplement: Figure 7—source data 1. [file elife-98016-fig7-data1.zip › Figure 7-Source Data 1/Figure 7D GFP SUN-unedited.tif]

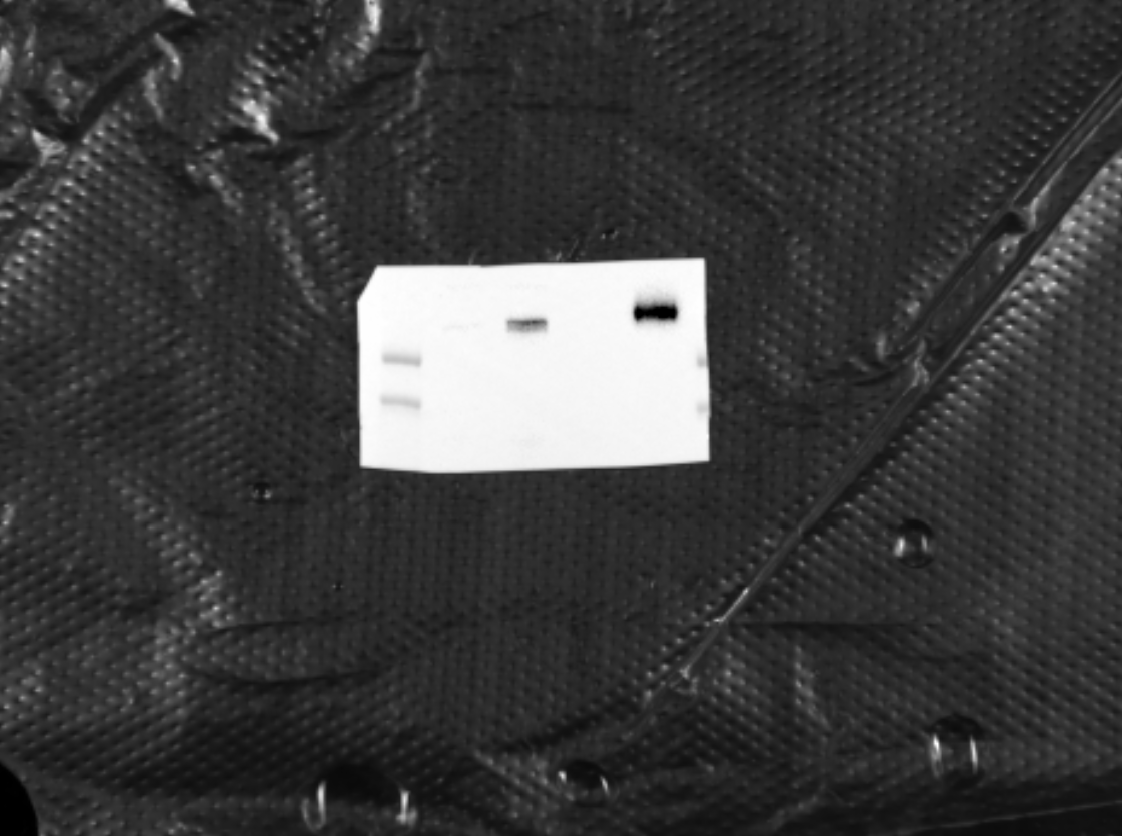

Supplement: Figure 7—source data 1. [file elife-98016-fig7-data1.zip › Figure 7-Source Data 1/Figure 7E FLAG CENTLEIN-unedited.tif]

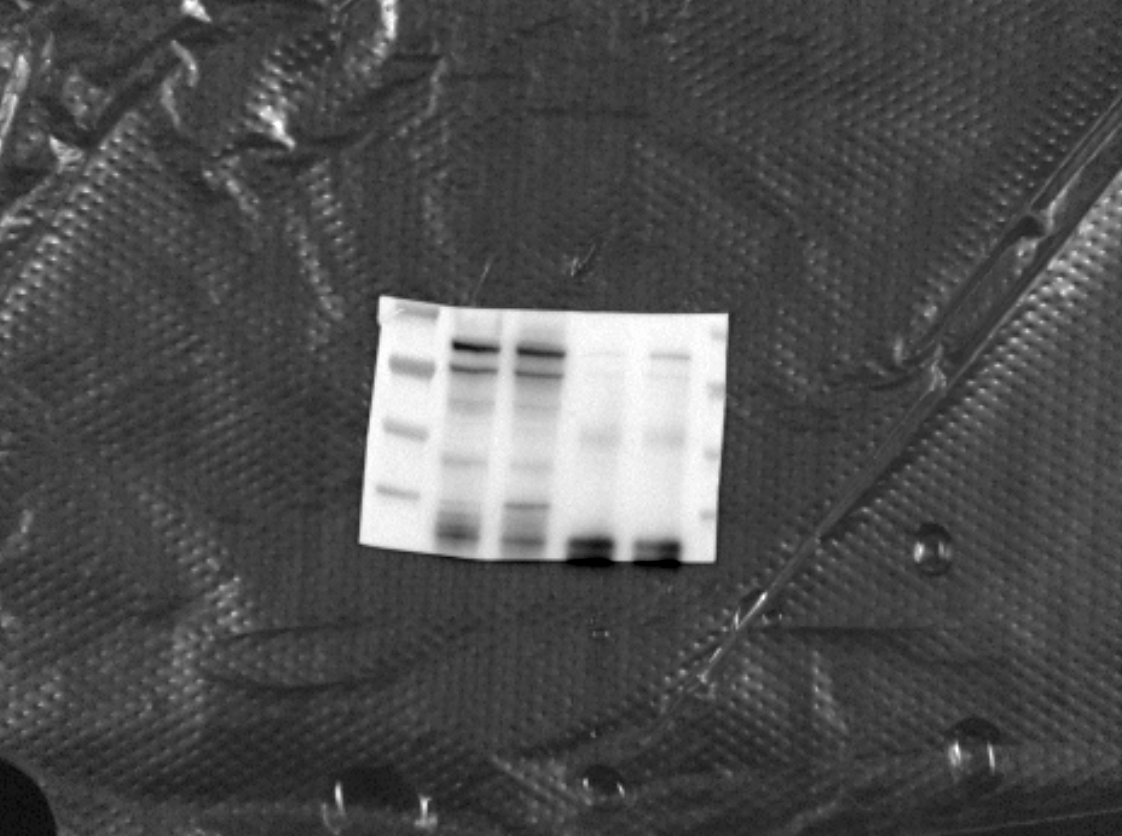

Supplement: Figure 7—source data 1. [file elife-98016-fig7-data1.zip › Figure 7-Source Data 1/Figure 7E GFP CCDC113-unedited.tif]

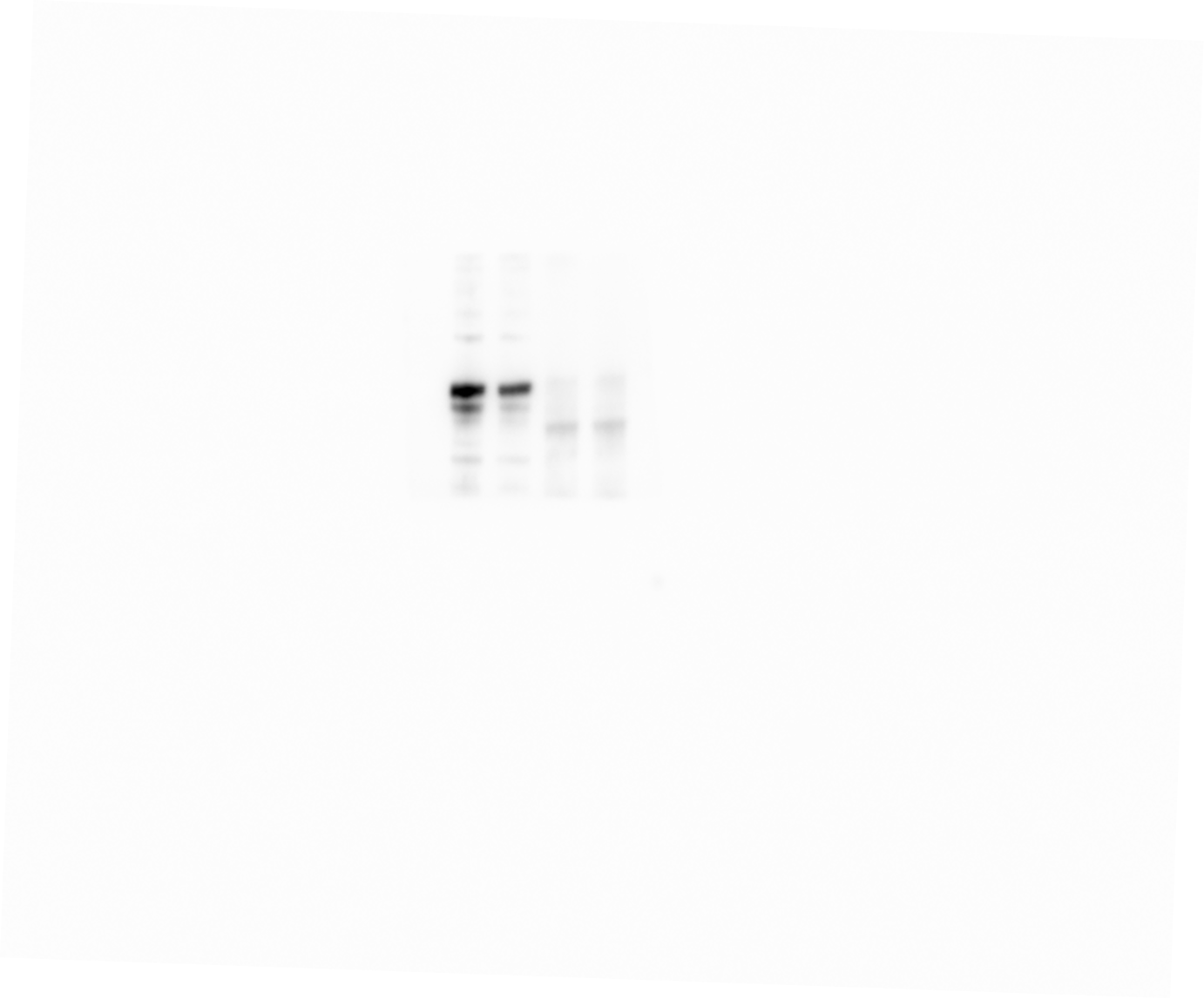

Supplement: Figure 7—source data 1. [file elife-98016-fig7-data1.zip › Figure 7-Source Data 1/Figure 7F FLAG SPATA6-unedited.Tif]

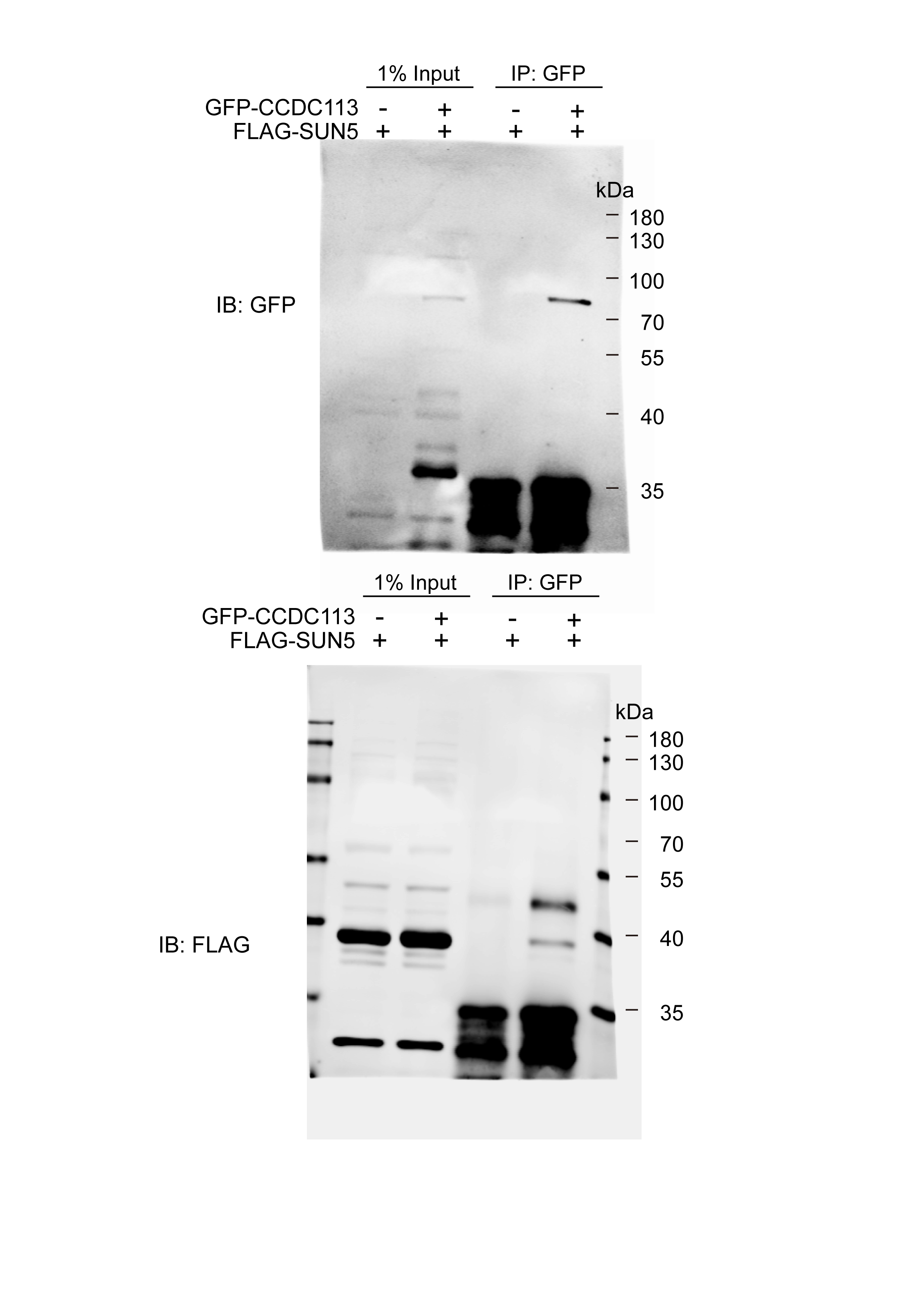

Supplement: Figure 7—source data 2. [file elife-98016-fig7-data2.zip › Figure 7-Source Data 2/Figure 7A labelled.tif]

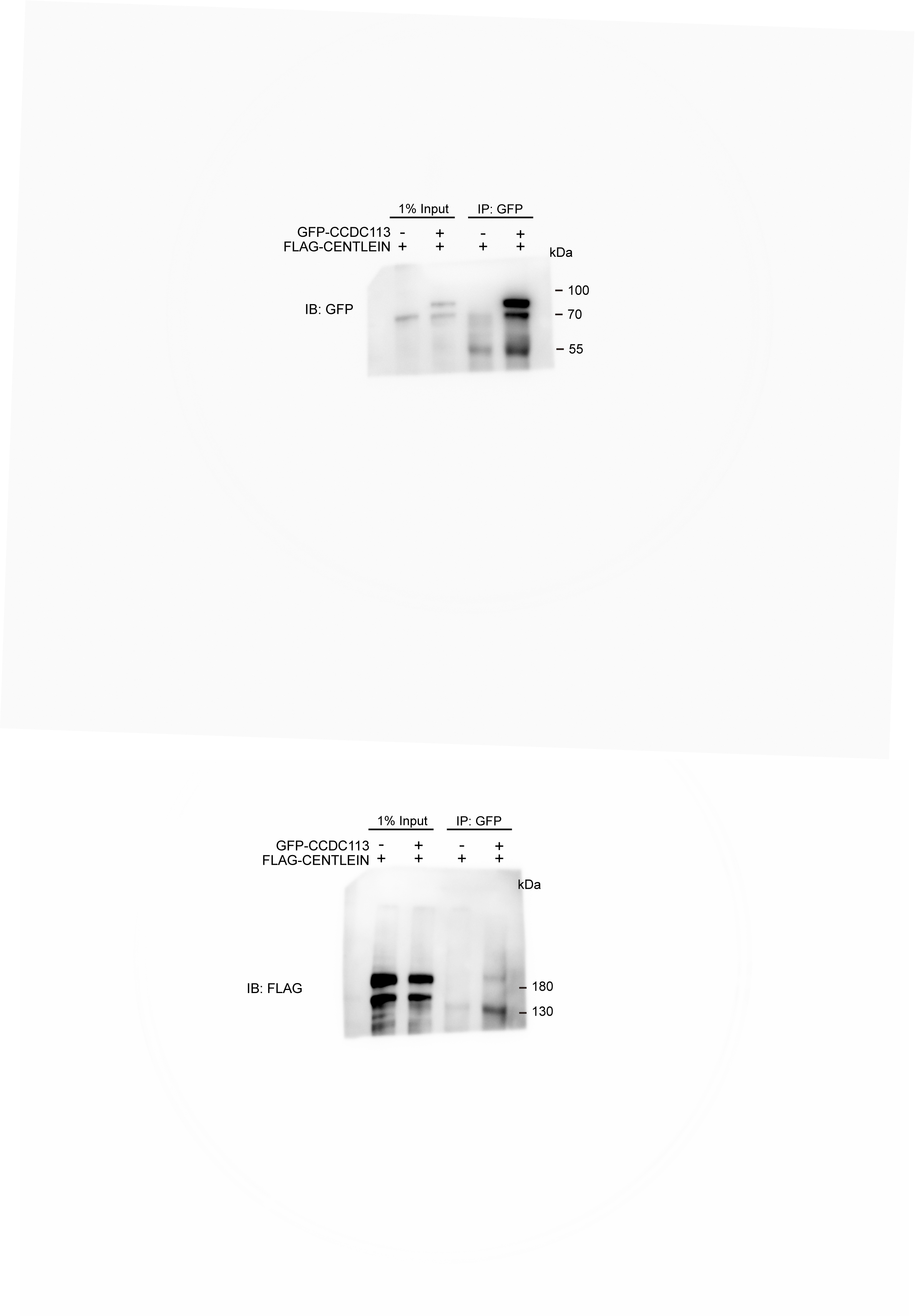

Supplement: Figure 7—source data 2. [file elife-98016-fig7-data2.zip › Figure 7-Source Data 2/Figure 7B labelled.tif]

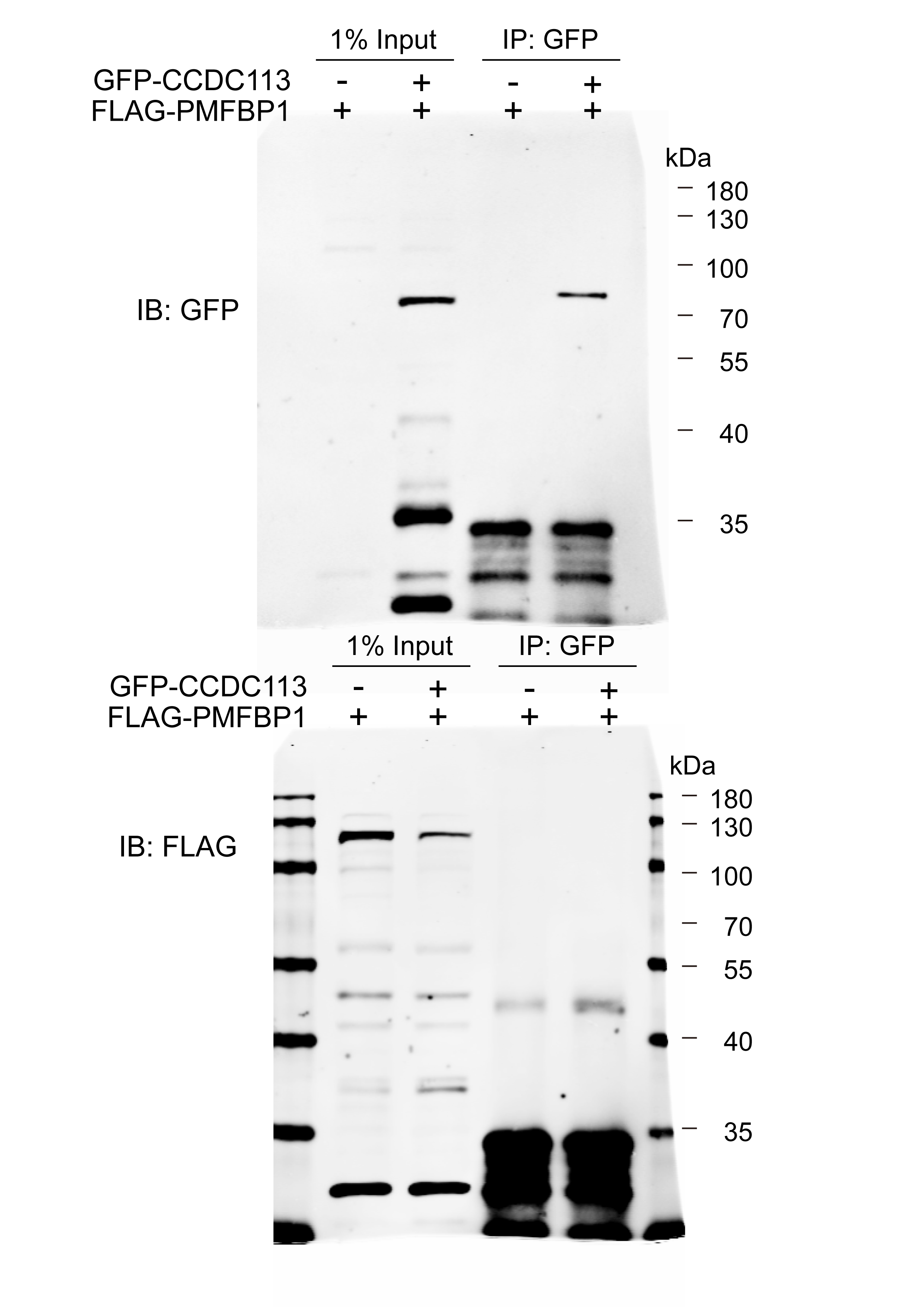

Supplement: Figure 7—source data 2. [file elife-98016-fig7-data2.zip › Figure 7-Source Data 2/Figure 7C labelled.tif]

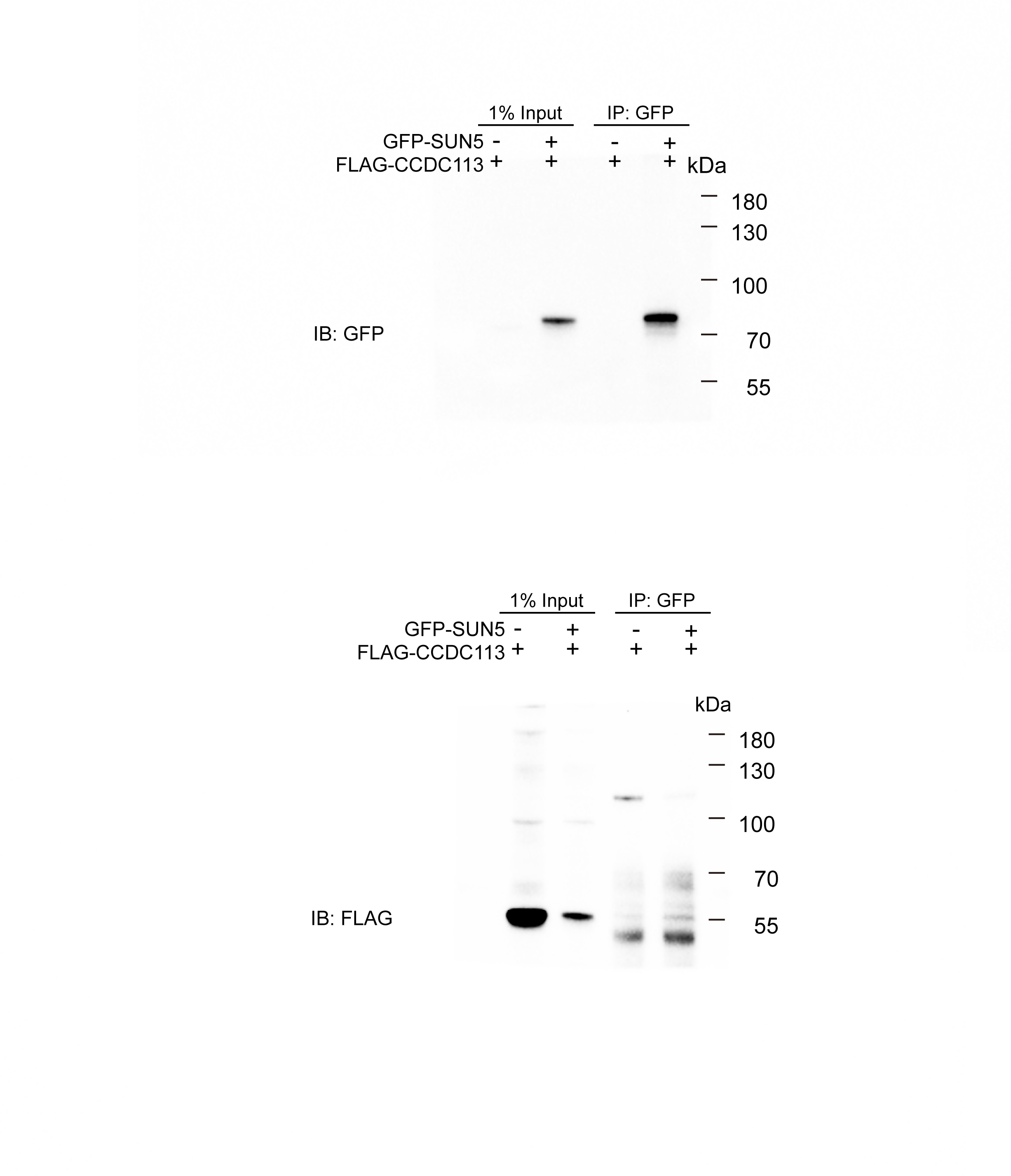

Supplement: Figure 7—source data 2. [file elife-98016-fig7-data2.zip › Figure 7-Source Data 2/Figure 7D labelled.tif]

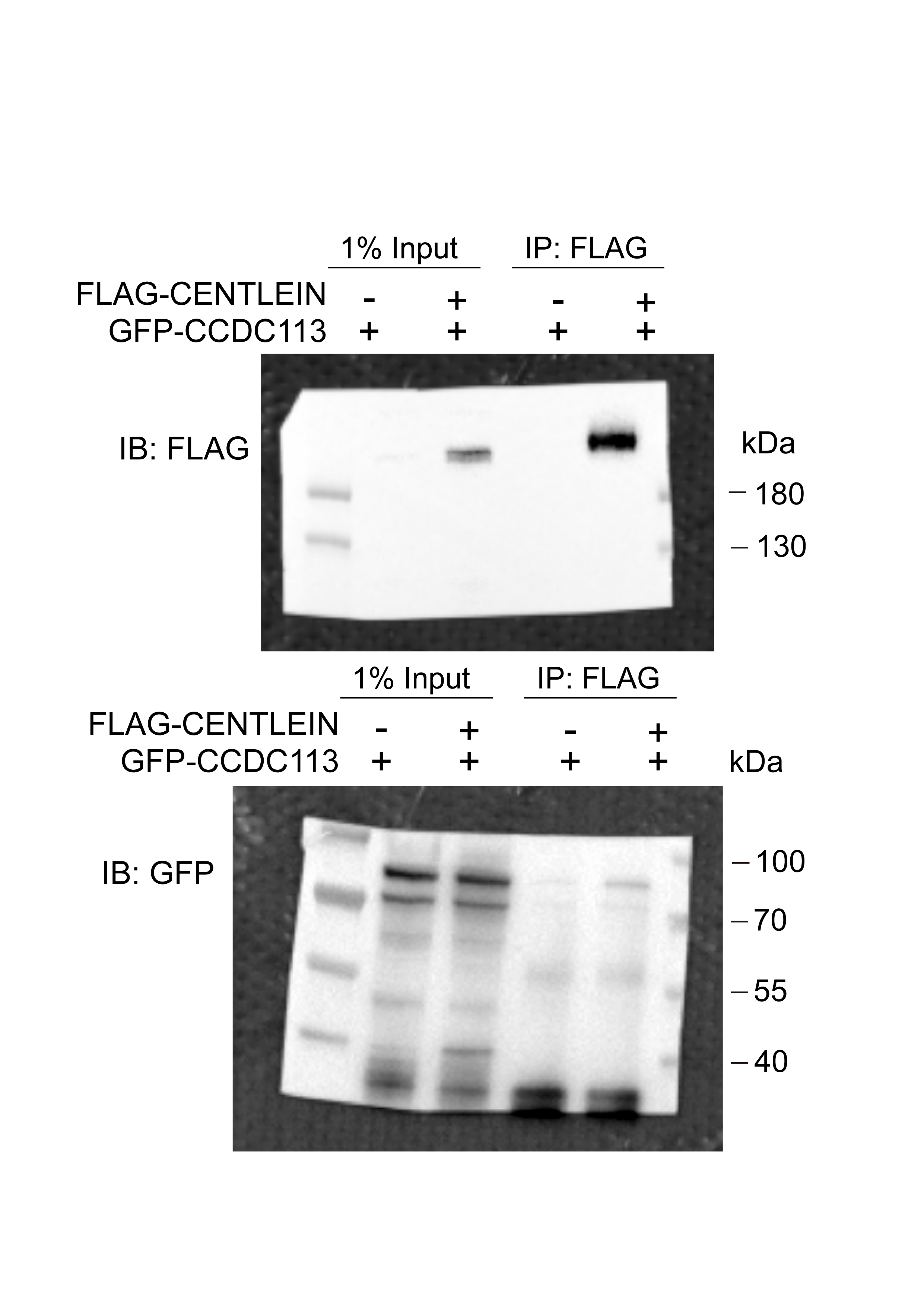

Supplement: Figure 7—source data 2. [file elife-98016-fig7-data2.zip › Figure 7-Source Data 2/Figure 7E labelled.tif]

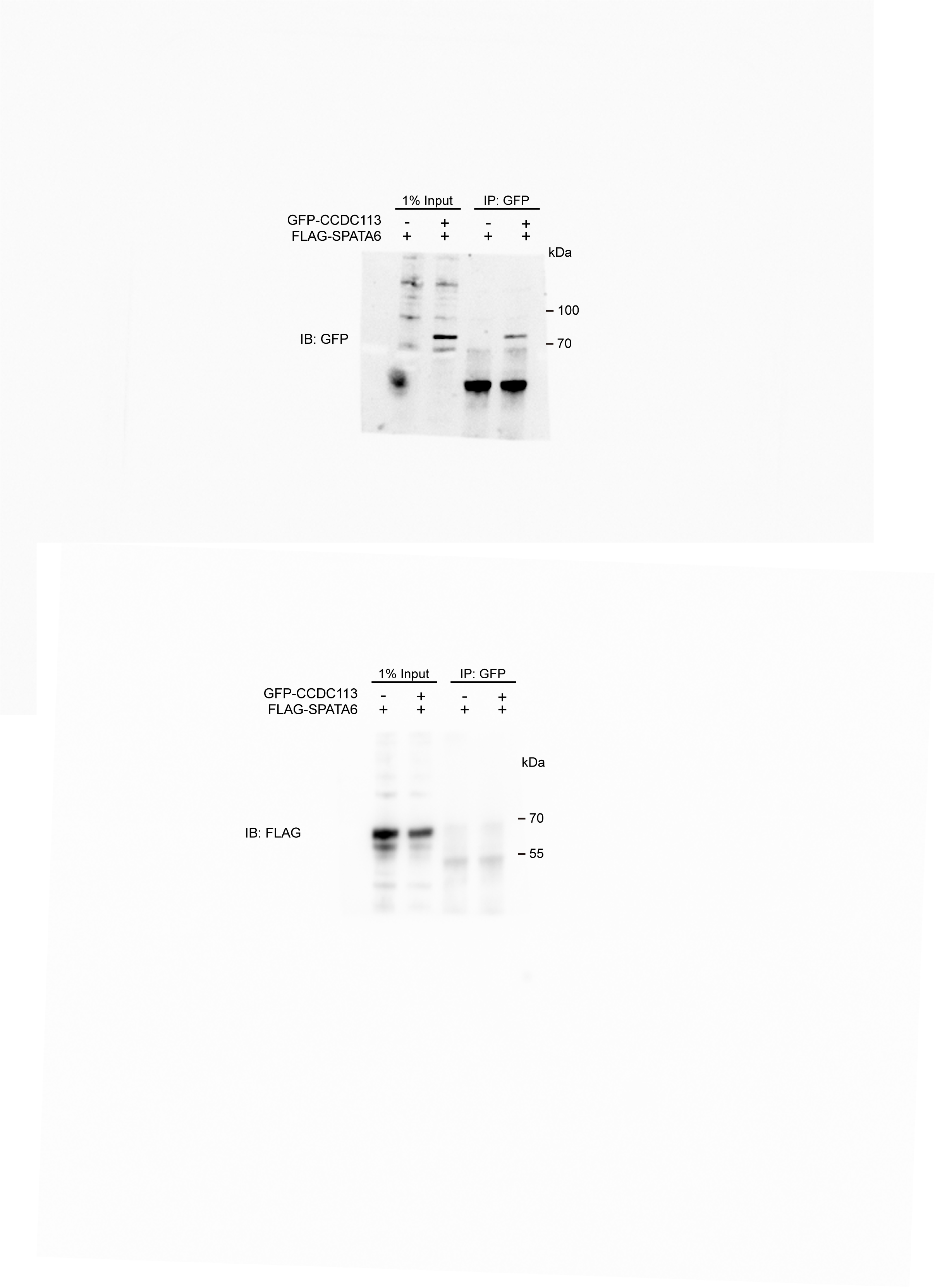

Supplement: Figure 7—source data 2. [file elife-98016-fig7-data2.zip › Figure 7-Source Data 2/Figure 7F labelled.tif]
